# Existing and potential infection risk zones of yellow fever worldwide: a modelling analysis

**DOI:** 10.1016/S2214-109X(18)30024-X

**Published:** 2018-02-02

**Authors:** Freya M Shearer, Joshua Longbottom, Annie J Browne, David M Pigott, Oliver J Brady, Moritz U G Kraemer, Fatima Marinho, Sergio Yactayo, Valdelaine E M de Araújo, Aglaêr A da Nóbrega, Nancy Fullman, Sarah E Ray, Jonathan F Mosser, Jeffrey D Stanaway, Stephen S Lim, Robert C Reiner, Catherine L Moyes, Simon I Hay, Nick Golding

**Affiliations:** a Big Data Institute, Li Ka Shing Centre for Health Information and Discovery, University of Oxford, Oxford, UK; b Department of Zoology, University of Oxford, Oxford, UK; c Institute for Health Metrics and Evaluation, University of Washington, Seattle, WA, USA; d Department of Infectious Disease Epidemiology, London School of Hygiene & Tropical Medicine, London, UK; e Harvard Medical School, Boston, MA, USA; f Boston Children's Hospital, Boston, MA, USA; g University of State of Rio de Janeiro, Maracana, Rio de Janeiro, Brazil; h World Health Organization, Infectious Hazard Management, Geneva, Switzerland; i Secretariat of Health Surveillance of the Ministry of Health of Brazil, Brazil; j Division of Pediatric Infectious Diseases, Seattle Children's Hospital/University of Washington, Seattle, WA, USA; k Quantitative & Applied Ecology Group, School of BioSciences, University of Melbourne, Parkville, VIC, Australia

## Abstract

**Background:**

Yellow fever cases are under-reported and the exact distribution of the disease is unknown. An effective vaccine is available but more information is needed about which populations within risk zones should be targeted to implement interventions. Substantial outbreaks of yellow fever in Angola, Democratic Republic of the Congo, and Brazil, coupled with the global expansion of the range of its main urban vector, *Aedes aegypti*, suggest that yellow fever has the propensity to spread further internationally. The aim of this study was to estimate the disease's contemporary distribution and potential for spread into new areas to help inform optimal control and prevention strategies.

**Methods:**

We assembled 1155 geographical records of yellow fever virus infection in people from 1970 to 2016. We used a Poisson point process boosted regression tree model that explicitly incorporated environmental and biological explanatory covariates, vaccination coverage, and spatial variability in disease reporting rates to predict the relative risk of apparent yellow fever virus infection at a 5 × 5 km resolution across all risk zones (47 countries across the Americas and Africa). We also used the fitted model to predict the receptivity of areas outside at-risk zones to the introduction or reintroduction of yellow fever transmission. By use of previously published estimates of annual national case numbers, we used the model to map subnational variation in incidence of yellow fever across at-risk countries and to estimate the number of cases averted by vaccination worldwide.

**Findings:**

Substantial international and subnational spatial variation exists in relative risk and incidence of yellow fever as well as varied success of vaccination in reducing incidence in several high-risk regions, including Brazil, Cameroon, and Togo. Areas with the highest predicted average annual case numbers include large parts of Nigeria, the Democratic Republic of the Congo, and South Sudan, where vaccination coverage in 2016 was estimated to be substantially less than the recommended threshold to prevent outbreaks. Overall, we estimated that vaccination coverage levels achieved by 2016 avert between 94 336 and 118 500 cases of yellow fever annually within risk zones, on the basis of conservative and optimistic vaccination scenarios. The areas outside at-risk regions with predicted high receptivity to yellow fever transmission (eg, parts of Malaysia, Indonesia, and Thailand) were less extensive than the distribution of the main urban vector, *A aegypti*, with low receptivity to yellow fever transmission in southern China, where *A aegypti* is known to occur.

**Interpretation:**

Our results provide the evidence base for targeting vaccination campaigns within risk zones, as well as emphasising their high effectiveness. Our study highlights areas where public health authorities should be most vigilant for potential spread or importation events.

**Funding:**

Bill & Melinda Gates Foundation.

## Introduction

The global spread of mosquito-borne viruses such as dengue, chikungunya, West Nile virus, and Zika virus during recent decades^1^ highlights the urgent need to better understand both contemporary arbovirus distributions and their potential to spread into new areas. Substantial outbreaks of yellow fever in Angola, the Democratic Republic of the Congo (DR Congo), and Brazil in the past 2 years,^2^ combined with the global distribution of the main urban vector *Aedes aegypti,* suggest that yellow fever has the potential to spread further internationally and increase its financial burden on health systems as well as its toll on population health.

Yellow fever is vaccine preventable, yet incomplete coverage means that the disease is still widely distributed in the tropics and subtropics of Latin America and Africa. Local transmission of yellow fever has never been reported in Asia, despite multiple opportunities for introduction and seemingly suitable ecological and climatic conditions.^3^

Multiple transmission cycles of yellow fever virus coexist with different mosquito genera serving as vectors in each cycle. The virus is principally maintained by a sylvatic transmission cycle involving non-human primate reservoirs, and human beings can sporadically be incidental hosts. When infected people introduce yellow fever virus into heavily populated areas with competent vector populations and insufficient vaccination, the virus is transmitted from person to person via *A aegypti*, and large-scale epidemics can occur. The spectrum of human clinical disease caused by yellow fever virus is broad, ranging from asymptomatic (unapparent) infections to fatal disease.^4^

**Research in context**

**Evidence before this study**

We searched PubMed on Dec 13, 2017, using the search terms “yellow fever” [All Fields] AND (“geograph*” [All Fields] OR “spati*” [All Fields]) without any date or language restrictions. This search returned 267 articles, of which seven contained information about the existing or potential geographical distribution of yellow fever risk. Five studies quantified yellow fever risk at national levels as well as at first or second administrative subnational units, or by ecological zone for a subset of countries; however, such results might mask considerable differences in yellow fever risk at more local levels. In 2006, Rogers and colleagues mapped yellow fever risk across both Africa and South America, as well as predicted climatic suitability for transmission outside these regions. This study analysed yellow fever data up to 2005, did not provide any estimate of spatial variation in disease incidence, or account for vaccination. An analysis of yellow fever data up to 2011 evaluated the impact of yellow fever vaccination in Africa, but the application of these findings might be limited in more recent years, and for other regions that have yellow fever epidemics (eg, Amazon basin). WHO has also done yellow fever risk assessments for high-risk and middle-risk African countries to identify regions most vulnerable to epidemics. To date, no study has quantified yellow fever risk or incidence across global risk zones at a high geospatial resolution, or evaluated potential for receptivity to yellow fever in areas outside current risk zones.

**Added value of this study**

We used 1155 records of human yellow fever virus infection from 1970 to 2016 to generate a novel model of incidence of yellow fever at a 5 × 5 km resolution for 47 countries across risk zones. We estimated potential receptivity to yellow fever transmission in all countries that are currently outside of defined risk zones. This work incorporated the most contemporary data on occurrence of yellow fever, and the chosen modelling approach allowed the inclusion of vaccination coverage rates, variation in disease reporting effort, mechanistic knowledge of suitability for vectors, distribution maps for reservoir and vector species, and a range of covariate data on land cover.

**Implications of all the available evidence**

Our work furthers understanding of the contemporary global distribution of yellow fever, and the potential for its introduction and establishment into new regions. These results provide a current evidence base to prioritise areas for vaccination and surveillance programmes in present risk zones (such as parts of Nigeria, the Democratic Republic of the Congo, and South Sudan), and highlight areas where public health authorities should be most vigilant for potential spread or importation events (including Central America, southeast Asia, and eastern Brazil).

The epidemiology of yellow fever on the African continent, where most cases and epidemics are reported, involves a mix of transmission cycles. In recent decades, almost all cases of yellow fever in Latin America arose from sylvatic cycles, with inter-human transmission only reported a handful of times since the early 1940s.^4^ Although eradication of yellow fever is not considered feasible because of its sylvatic reservoir, outbreak control is achievable because of the availability of a safe, low cost, and effective vaccine. Since the vaccine became available in 1937, the combination of vaccination and vector control strategies has led to a notable reduction in disease burden at some locations and times.^5^

The outbreak that started in Luanda, Angola, in December, 2015, developed into the largest and most widespread outbreak of yellow fever reported in Africa in more than 20 years.^6^ In March, 2016, several unvaccinated Chinese workers with yellow fever virus infection returned to China from the outbreak zone in Angola, which, alongside the depletion of global emergency vaccine stockpiles, raised concerns that yellow fever would gain an uncontrollable foothold in east and southeast Asia.^7^ Fortunately, these importation events occurred in Beijing, Shanghai, and Fujian, where *A aegypti* is not established, and no secondary cases resulting from local transmission were detected.^8^

Informing vaccination and other control strategies for yellow fever requires an improved evidence base for identifying the most vulnerable populations. Risk mapping for yellow fever occurrence in areas at risk of transmission has previously been done at national and subnational levels,^5^, ^9^, ^10^ but the distribution of the disease within these areas remains poorly characterised. For areas outside the defined risk zones,^9^ the most recent efforts to identify populations at risk have been based on the distribution of *A aegypti*.^11^ Although presence of the vector is necessary for transmission, many other factors contribute towards the establishment and maintenance of transmission.^12^

In this study, we modelled subnational variation in risk of yellow fever across all risk zones and predicted receptivity to yellow fever virus transmission outside risk areas, using a model that is based on yellow fever infection data, vaccination coverage rates, and a range of environmental and biological covariates.

## Methods

### Overview

We collated and georeferenced reports of symptomatic yellow fever virus infection in people for the 47-year period from 1970 to 2016. Records before 1970 were not included because we aimed to map the contemporary spatial distribution of yellow fever, and this timepoint coincides with the re-infestation of Latin America with *A aegypti* following 50 years of eradication efforts,^13^ and the cessation of organised large-scale yellow fever vaccination activities in west Africa and central Africa.^5^ We fitted a Poisson point process boosted regression tree (BRT) model to the database of yellow fever disease reports along with a range of environmental and socioeconomic variables postulated to influence yellow fever transmission. By explicitly accounting for population density, spatial variation in disease reporting rates, and vaccination coverage, we estimated the relative risk of apparent infection for each 5 × 5 km grid square across risk zones (47 countries across the Americas and Africa). To map within-country variation in annual incidence, we used previously published national case estimates from the Global Burden of Diseases, Injuries, and Risk Factors Study 2015 (GBD 2015).^14^ The incidence map was averaged over 47 years to visualise spatial variation without the large temporal fluctuations seen for yellow fever. Finally, the fitted model for relative infection risk was used to predict areas of receptivity to yellow fever virus transmission beyond contemporary risk zones. A schematic overview of the process we followed is provided in the appendix (p 2).

### Dataset

**Figure 1:**
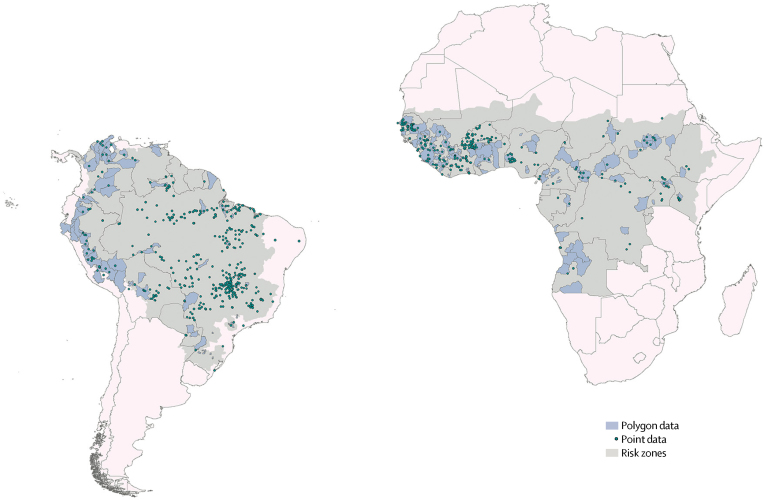
Yellow fever occurrence data provided to the model Polygon and point locations of reports of human yellow fever virus infection from 1970 to 2016. Points represent yellow fever virus infections reported in locations smaller than 5 × 5 km in area, where only the latitude and longitude of the site were recorded. Polygons represent yellow fever virus infections reported in locations larger than 5 × 5 km in area, which were assigned an administrative area code (eg, province or district). Grey areas represent contemporary risk zones as defined by Jentes and colleagues.^9^

We assembled a database of locations where at least one laboratory-confirmed symptomatic human infection of yellow fever virus had been reported in any given year. This information was extracted from various sources including online sources, peer-reviewed literature, and WHO reports. Data from online sources were collated by the automated HealthMap system, as described elsewhere.^15^ Spatial coordinates were assigned to each reported site of infection, only including sites where infections were diagnosed by serological, PCR-based, or other genetic detection techniques. For locations smaller than 5 × 5 km in area (points), only the latitude and longitude were recorded. The remaining sites (polygons) were assigned an administrative area code (eg, for a province or district). The final dataset included 1155 records, comprising 751 point locations and 404 polygon locations (figure 1). Details on the assembly of the occurrence dataset can be found in the appendix (pp 2–3).

### Vaccination coverage and population density data

To account for the effects of yellow fever vaccination in our temporally static model of yellow fever infection risk, we required an estimate of the average susceptible population size across the study time period. To calculate this temporal average, we used vaccination coverage estimates for every 5 years from 1970 to 2016 (for which details can be found elsewhere),^16^ and a combination of WorldPop Project,^17^ Gridded Population of the World,^18^ and UN World Population Prospects human population density data.^19^ Details of this procedure are described in the appendix (pp 4–5).

### Model explanatory covariates

Ten 5 × 5 km gridded data surfaces of a range of environmental and biological factors thought to affect transmission of yellow fever virus were used as potential explanatory covariates (table).^20^, ^21^, ^22^, ^23^, ^24^, ^25^ Predictive species distribution maps of suspected reservoir species of non-human primates in Africa and Latin America were generated using a previously described approach,^26^ and were included in the model as biological covariates. See appendix (pp 6–12) for further details regarding the construction of each covariate data surface and for plots of each surface.

**Table:** Explanatory covariates included in the analysis

	**Data source**
Evergreen broadleaf forest, urban and built up, and cropland mosaics land cover classes (proportional)	MODIS land cover product^25^
Elevation	Shuttle Radar Topography Mission^20^
Tasselled cap wetness, a measure of surface moisture (mean)	Gap-filled MODIS satellite data^21^, ^22^
Enhanced vegetation index (mean)	Gap-filled MODIS satellite data^21^, ^22^
*Aedes aegypti* temperature suitability index	Brady et al (2014)^23^
*A aegypti* habitat suitability	Kraemer et al (2015)^24^
Predictive species distribution of suspected reservoir non-human primates	Prepared for this project (appendix pp 9–12)

MODIS=moderate resolution imaging spectroradiometer.

### Modelling approach

We fitted an inhomogeneous Poisson point process model to the dataset of yellow fever disease reports using a BRT model to characterise the expected number of observations per unit area. By explicitly accounting for human population density, vaccination coverage rate, and variable reporting effort, this model estimated the incidence of apparent infections in susceptible individuals (which can be interpreted as the individual risk of apparent infection), to within a constant of proportionality at each 5 × 5 km grid square across risk zones.^9^ The fitted model was then used to estimate receptivity to yellow fever transmission in areas outside contemporary risk zones. See appendix (pp 13–16) for details of model specification and fitting.

### Calibration of raw model outputs

By considering the size of the susceptible population within each 5 × 5 km grid square (based on vaccination coverage levels in 2016), and applying continental calibration factors, we estimated the average annual number of cases across risk zones (averaged over the 47-year study period). Calibration factors were calculated using the average annual number of cases (from 1990 to 2015) estimated by GBD 2015^14^ and a generalised linear model—ie, we used a continental-scale estimate of incidence to calculate case detection rate, which is not identifiable from occurrence data alone. Details of the calibration procedure are described in the appendix (pp 17–18). We generated calibration factors of 155·15 (lower bound 44·27, upper bound 405·99) for Africa, and 38·95 for South America (lower bound 10·32, upper bound 108·20). The mean calibration factors were applied to the raw model output to map average annual incidence and case numbers.

### Model validation

The model's predictive performance within risk zones was assessed by spatially stratified ten-fold cross-validation and by calculating the predictive deviance of the model, which is the mismatch between the predicted number of occurrence records and the number observed. Cross-validation was done for each bootstrap of the model, resulting in a bootstrapped estimate of the model's predictive capacity. Details of the validation procedures and results are provided in the appendix (p 16).

### Cases averted by vaccination

We estimated the average annual number of cases of yellow fever per pixel using estimated vaccination coverage rates in 2016, based on three vaccination targeting scenarios for historical campaigns: conservative (untargeted, biased); untargeted, unbiased; and optimistic (targeted).^16^ To estimate the average annual number of cases of yellow fever averted within risk zones by 2016 vaccination levels, the outputs of conservative and optimistic cases per pixel were compared with a fourth scenario assuming 100% of the population in 2016 were susceptible to yellow fever virus infection (ie, vaccination coverage rates assumed to be zero). Because our model estimates the relative risk of yellow fever infection from 1970 to 2016, and therefore assumes a constant rate of yellow fever infection through time, these values represent the cases averted by 2016 vaccination levels given the average annual infection rate, not the specific infection rate in 2016, and assuming constant exposure to a source of yellow fever virus infection.

### Role of the funding source

The funders of the study had no role in study design, data collection, data analysis, data interpretation, or writing of the report. The corresponding author had full access to all the data in the study and had final responsibility for the decision to submit for publication.

## Results

**Figure 2:**
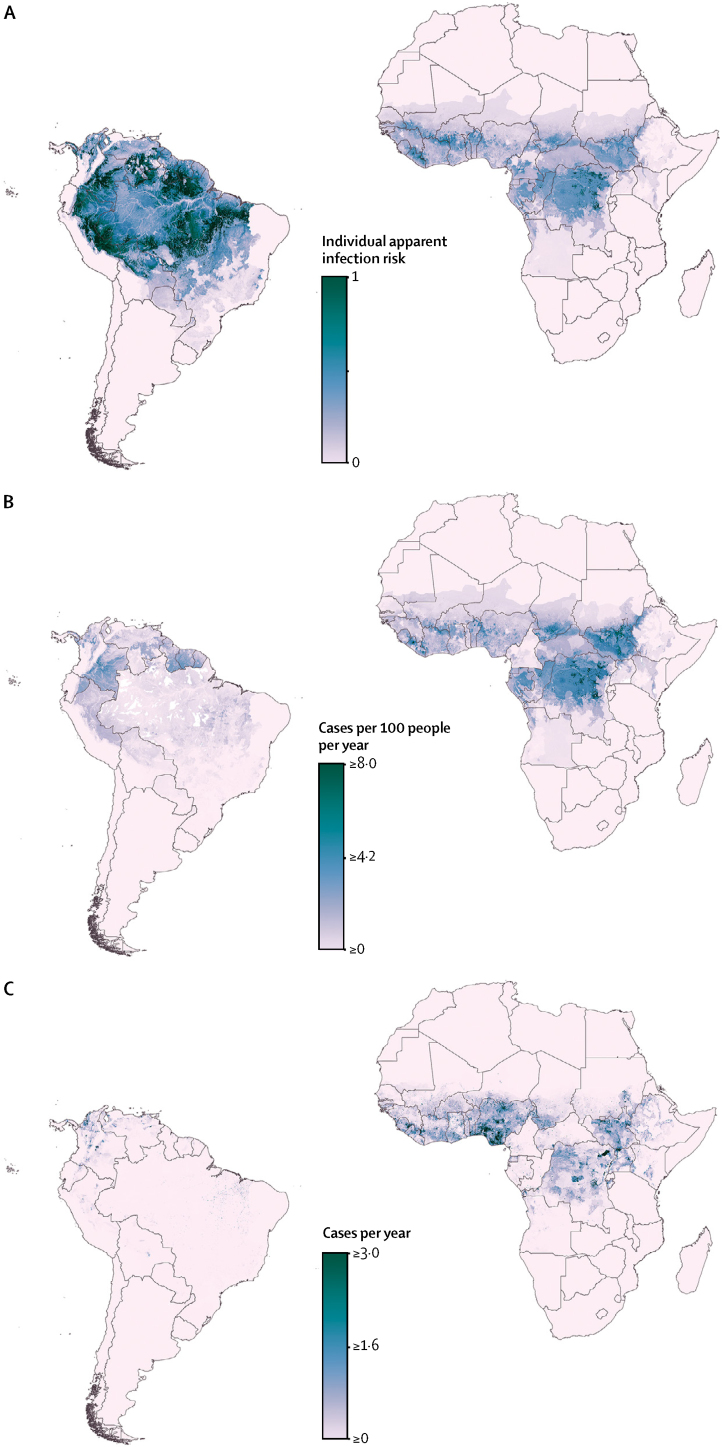
Predicted distribution of yellow fever within contemporary risk zones (A) Predicted spatial variation in individual risk of yellow fever virus infection. (B) Predicted average annual incidence of yellow fever (99·8% of grid squares within the disease's range were predicted to have fewer than eight cases per 100 people per year, the highest predicted value was 20 cases per 100 people per year). (C) Average annual numbers of yellow fever cases (99·5% of grid squares within the disease's range were predicted to have fewer than three cases per year, the highest predicted value was 109 cases per year). Continental calibration factors have been applied to the outputs in B and C, calculated from Global Burden of Diseases, Injuries, and Risk Factors Study 2015 estimates of national incidence averaged from 1990 to 2015 (see full details in the Methods and appendix pp 17–18),^14^ and use population vaccination coverage rates achieved in 2016. All predictions were restricted to areas within the contemporary risk zones as defined by Jentes and colleagues^9^ and averaged over the 47-year study period from 1970 to 2016.

**Figure 3:**
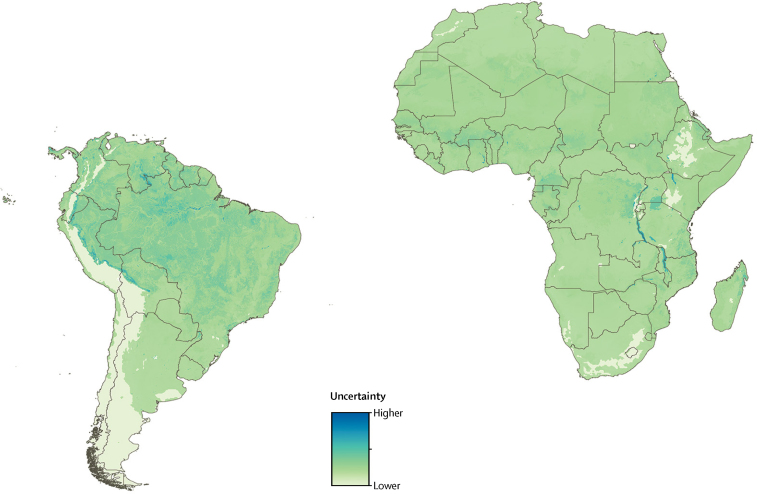
Map of model uncertainty Estimated pixel-wise uncertainty in spatial predictions of individual risk of apparent yellow fever virus infection, based on standard deviation values calculated for each pixel across the model ensemble.

Our model predicted substantial international and subnational spatial variation in individual infection risk for yellow fever (figure 2). This risk can be interpreted as the rate at which susceptible individuals are expected to acquire a symptomatic yellow fever virus infection in a given location. The underlying risk for yellow fever can therefore be high in locations with negligible population density or high vaccination coverage. Areas with highest predicted individual infection risk within Latin America included the Amazonian regions of Brazil (particularly Pará, Acre, and Roraima states), Bolivia, Colombia, Ecuador, French Guiana, Guyana, Peru, Suriname, and Venezuela. Within the African risk zone, the areas with highest predicted individual infection risk included several locations within Burkina Faso (Cascades and Sud-Ouest), Cameroon (Centre, Est, Littoral, Sud-Ouest, and Nord), Côte d'Ivoire (Savanes, Zanzan, and Montagnes), DR Congo (Sankuru, Maï-Ndombe, Équateur, Maniema, Mongala, Tshuapa, Kwilu, Nord-Ubangi, Bas-Uele, Haut-Uele, Ituri, and Tshopo), Ghana (Northern and Upper West), Liberia (Bong, Gbarpolu, Grand Gedeh, Lofa, and Nimba), Republic of the Congo (Sangha), Sierra Leone (Eastern), Mali (Sikasso), and South Sudan (Jonglei). Model uncertainty in spatial predictions of individual infection risk for yellow fever is shown in figure 3; there is low uncertainty in predictions in most areas.

Considered together, the maps of individual infection risk, average annual incidence, and average annual cases (figure 2) highlight the effects of vaccination on incidence of yellow fever. For example, populations of Brazil, Bolivia, Venezuela, Guinea, Cameroon, and Togo have high predicted rates of underlying risk of yellow fever, but low predicted annual incidence, on the basis of vaccination coverage levels achieved by 2016 (appendix p 5).^16^ Conversely, they also suggest that many populous regions, with patchy or limited vaccination coverage, have a high exposure risk to yellow fever virus infection and are poorly protected against transmission. For example, areas with the highest predicted average annual cases included large parts of DR Congo (Sankuru, Maï-Ndombe, Équateur, Mongala, Tshuapa, Kwilu, Nord-Ubangi, Sud-Ubangi, Bas-Uele, Haut-Uele, and Tshopo), Nigeria (South-West, South-East, South-South, and North-Central zones), and South Sudan (Jonglei, Eastern Nile, Western Nile, Terekeka, Yei River, and Jubek), as well as smaller areas within Côte d'Ivoire (Montagnes), Sierra Leone (Eastern), Ghana (Ashanti and Northern), Mali (Sikasso), Ethiopia (Amhara region and Southern Nations, Nationalities, and Peoples [SNNP] region), and Uganda (Adjumani). For most of these locations, vaccination coverage in 2016 was estimated to be less than the 60–80% recommended by WHO to prevent outbreaks.^10^

Using the total average annual cases estimated by our model, we calculated that globally, between 94 336 and 118 500 cases of yellow fever are averted each year by vaccination coverage rates achieved in 2016 (based on conservative and optimistic vaccination coverage scenarios, respectively). These numbers represent 33% to 42% of total predicted cases if vaccination coverage was zero across risk zones. The global totals represent between 84 385 and 99 840 cases averted on the African continent (33% to 39% of total predicted cases) and between 9951 and 18 660 cases within Latin America (36% to 68% of total predicted cases).

**Figure 4:**
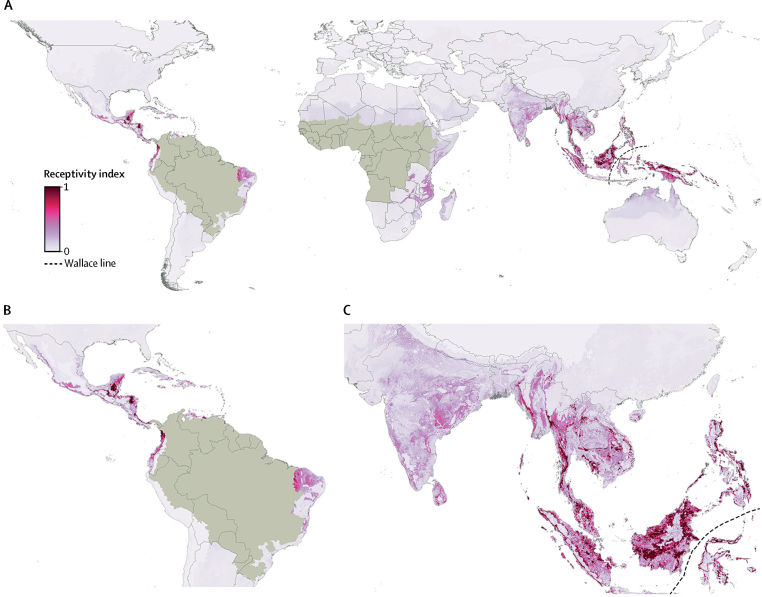
Predicted receptivity to yellow fever transmission outside contemporary risk zones Receptivity to yellow fever transmission in areas outside the contemporary risk zones, as defined by Jentes and colleagues^9^ and shown in light brown. No species known to be potential hosts of yellow fever virus persist in areas east of the Wallace line (faunal boundary).

Outside contemporary risk zones, we predict high receptivity to yellow fever virus transmission across much of southeast Asia, where yellow fever has never been reported, including large areas of Cambodia, India, Indonesia, Laos, Malaysia, Myanmar, Papua New Guinea, the Philippines, Thailand, and Vietnam (figure 4). High receptivity is also predicted in several countries within Central and South America, contiguous with risk zones. Historically, yellow fever has been reported in many of these countries, such as Costa Rica, Guatemala, Honduras, Nicaragua, and Mexico.^27^ We predict low receptivity to yellow fever virus transmission in several locations where *A aegypti* is known to be present, for example southern China and southern USA.

## Discussion

The high resolution maps of risk and incidence of yellow fever produced in this study highlight the success of vaccination in reducing incidence in high-risk regions, including Brazil, Cameroon, and Togo. The maps also identify areas with high predicted average annual case numbers, where vaccination coverage in 2016 was less than the recommended threshold to prevent outbreaks, such as large parts of Nigeria, DR Congo, and South Sudan. These maps provide an evidence base to prioritise areas for vaccination and vector control programmes in current risk zones. Receptivity to yellow fever virus transmission in areas outside risk zones was also mapped, including areas where yellow fever has been controlled, such as Central America, or has never persisted, such as southern Asia. Our findings highlight areas where public health authorities should be most vigilant for potential spread or importation events. Furthermore, the areas of high receptivity near the edge of the current risk zone on the southeast coast of Brazil (states of Bahia, Minas Gerais, São Paulo, Espírito Santo, and Rio de Janeiro) reflect a need to geographically expand the existing risk limits because locally acquired cases have recently been reported in this area.^28^

Rogers and colleagues^3^ used discriminant analysis techniques to predict areas of climatic suitability for yellow fever within Asia, including eastern Thailand and parts of Malaysia and Indonesia. Building on their analysis, our model predicts more extensive receptivity to yellow fever virus transmission within these countries, as well as other parts of the continent. We have extended the work of Rogers and colleagues by fitting, to the most recent occurrence data, a model that has been demonstrated to perform well, accounts for bias in disease reporting rates, and incorporates vaccination coverage rates. Furthermore, our model includes mechanistic knowledge of habitat suitability for vectors, a range of covariate data on land cover, and distribution maps for reservoir and vector species. To our knowledge, this work is the first example of a disease niche modelling approach that incorporates vaccination coverage rates. Our results for Africa (33–39% of cases averted from 1970 to 2016) add to a previous analysis that estimated that vaccination campaigns averted the number of cases by 22% to 31% from 1987 to 2011.^5^ Our study includes more recent (and historical) occurrence data, and overall highlights the differences between Latin America and Africa.

We included distributions of *A aegypti* and non-human primate hosts in our model, because countries where both exist are thought to be most vulnerable to the introduction and establishment of yellow fever virus.^12^ Importation of the virus by an infected traveller could initiate an urban epidemic and, if suitable vector and reservoir species are present, subsequently trigger an enzootic transmission cycle, leading to a long-term infection risk for the local population.^7^ The present ease and frequency of international travel, compounded by poor enforcement of travel vaccination requirements, low levels of vaccination coverage in many at-risk regions,^16^ and existing vaccine shortages, raise serious concerns of introduction of yellow fever virus into naive human populations, particularly in southern Asia.^8^

Yellow fever has both urban and sylvatic transmission cycles, and the spatial and temporal distribution of cases fluctuates substantially. The periodicity of yellow fever epidemic activity is driven in part by the rapid depletion and slow replenishment of susceptible hosts, as illustrated by upsurges at irregular intervals in parts of west and east Africa. Our model did not attempt to capture temporal and spatial spikes in cases of yellow fever; the focus was on predicting spatial variation in the underlying risk of infection. Our outputs represent the period from 1970 to 2016, averaged over the large fluctuations that occur. Thus, the results might smooth over important secular trends across time, and our estimated incidence for a particular location will be different from the 2016 incidence, or even a 5-year average. Indeed, the five most recently reported outbreaks of yellow fever began in areas of variable predicted risk. Additionally, since all reports of yellow fever virus infection in human beings were included in the model, irrespective of whether infection was the result of a sylvatic, intermediate, or urban transmission cycle, the model predicts apparent infection risk from any transmission cycle. The BRT model is capable of encompassing different relations in the data arising from these distinct transmission cycles, with the given covariates.

Our model predicts receptivity to yellow fever virus transmission based on the relation between locations where yellow fever has been reported from 1970 to 2016, and the values of environmental and biological covariates at those locations. For this reason, predictions of high receptivity in southeast Asia should be interpreted with caution. Potential variables that distinguish current risk zones in Africa and the Americas from *A aegypti*-inhabited areas of southeast Asia might be missing from our analysis. Indeed, most theories that have been used to explain the absence of yellow fever in Asia involve biological factors rather than climatic or environmental ones. These factors include cross-protection from other flaviviruses, lower competence of local populations of *A aegypti*, and competition between other flaviviruses within mosquito cells,^29^ none of which are included in our model. The strength of the BRT approach is in its predictive power and ability to fit complex non-linear functions, with the given covariates. It is not possible to use this type of analysis to identify causal associations between the covariates and suitability for disease transmission. It would not be appropriate to make further inferences as to why the pattern of high receptivity predicted by our model is more contracted than the distribution of yellow fever's main urban vector, *A aegypti,* or to further speculate on reasons for the disease's absence from Asia to date. Our work, however, highlights areas for future improvement in our understanding of yellow fever ecology.

Our study involved multiple stages of analysis, each containing potential sources of bias and uncertainty, much of which was difficult to account for or quantify. Each stage of the analytical process involved fitting a model to an independent, fixed dataset. Since each of these models was fitted to fixed data, not the output of previous models, it was not meaningful to propagate uncertainty through these steps. However, there were some steps where it was important to account for or report uncertainties. In fitting the model of risk for apparent yellow fever virus infection, uncertainty in the spatial locations of reported cases was propagated through the model via Monte Carlo simulation. Similarly, uncertainty in the final step of the analytical process—calibrating spatial predictions of yellow fever risk against GBD 2015 estimates of annual cases—was estimated and reflected in reported results.

High-quality spatial data on yellow fever are lacking, largely because of diagnostic complexity and limitations of health-care systems in many affected countries. Our occurrence dataset included human yellow fever virus infections confirmed via both genetic and serological diagnostic methods. As a result of cross-reactivity among flaviviruses, the precision of serological diagnostic techniques is limited, particularly when considering historical records. Additionally, we assumed that estimates of vaccination coverage, combined with data on population size, were proportional to the number of people susceptible to yellow fever virus infection. Translating maps of unvaccinated individuals into maps of susceptible people is complicated by the acquisition of immunity via natural infection, which is difficult to quantify. Modelling efforts will be improved as the volume and quality of geographical data on yellow fever increases, ideally using diagnostics that distinguish past infection from vaccination, and from other flavivirus infections. For now, the work presented here provides the best available evidence base for identifying populations most vulnerable to yellow fever.

To develop the most cost-effective vaccination strategies that prevent outbreaks and minimise adverse events, vaccination policy makers require a clear understanding of geographical disease risk. Within risk zones, our model improves understanding of geographical risk and comes at a time when current policies may require re-evaluation in view of the demonstrated inadequacy of emergency stocks and surge capacity of vaccine manufacture to meet the needs of the recent outbreak in Angola.^7^ Because the vector of urban yellow fever transmission also transmits dengue virus, chikungunya virus, Zika virus, and other human pathogens, the work presented here can also inform integrated vector control strategies.^30^ Beyond risk zones, our map of receptivity identifies areas where governments should consider the need for travellers to or from countries endemic for yellow fever to be vaccinated, under the framework of the International Health Regulations, as well as assessing their capabilities for early detection and intervention. If local transmission was to occur in these locations, the map additionally provides an evidence base for predicting and limiting its spread.
