# Supplementary material for: Existing and potential infection risk zones of yellow fever worldwide: a modelling analysis
Source: Lancet Glob Health. 2018 Feb 2;6(3):e270–8. doi: 10.1016/S2214-109X(18)30024-X (PMC5809716; doi:10.1016/S2214-109X(18)30024-X)
Supplement: Supplementary appendix [file mmc1.pdf]

THE LANCET
Global Health

Supplementary appendix

This appendix formed part of the original submission and has been peer reviewed. We post it as supplied by the authors.

Supplement to: Shearer FM, Longbottom J, Browne AJ, et al. Existing and potential infection risk zones of yellow fever worldwide: a modelling analysis. *Lancet Glob Health* 2018; published online Jan 18. http://dx.doi.org/10.1016/S2214-109X(18)30024-X.

Appendices to "Existing and potential infection risk zones of yellow fever worldwide: a modelling analysis"

Freya M Shearer[1], Joshua Longbottom[1], Annie J Browne[1], David M Pigott[2], Oliver J Brady[3], Moritz UG Kraemer[4,5,6], Fatima Marinho[7], Sergio Yactayo[8], Valdelaine EM de Araújo[9], Aglaêr A da Nóbrega[9], Nancy Fullman[2], Sarah E Ray[2], Jonathan F Mosser[2,10], Jeffrey D Stanaway[2], Stephen S Lim[2], Robert C Reiner Jr[2], Catherine L Moyes[1], Simon I Hay[1,2], Nick Golding[11]

[1]Big Data Institute, Li Ka Shing Centre for Health Information and Discovery, University of Oxford, Oxford OX3 7LF, United Kingdom
[2]Institute for Health Metrics and Evaluation, University of Washington, 2301 5th Avenue, Suite 600, Seattle, WA, 98121, United States of America
[3]Department of Infectious Disease Epidemiology, London School of Hygiene and Tropical Medicine, London, WC1E 7HT, United Kingdom
[4]Department of Zoology, University of Oxford, New Radcliffe House, Radcliffe Observatory Quarter, 6GG, Woodstock Rd, Oxford OX2, United Kingdom
[5]Harvard Medical School, 25 Shattuck St, Boston, MA 02115, United States of America
[6]Boston Children's Hospital, 300 Longwood Ave, Boston, MA 02115, United States of America
[7] University of State of Rio de Janeiro, Maracana, Rio de Janeiro 20550-900, Brazil
[8]World Health Organization, Infectious Hazard Management (IHM), 20 Av Appia, 1211 Geneva 27. Switzerland
[9]Secretariat of Health Surveillance of the Ministry of Health of Brazil, SRTVN 701, Via W5 Norte, Ed. PO700, 6º andar, CEP 70723-040, Brazil
[10]Division of Pediatric Infectious Diseases, Seattle Children's Hospital/University of Washington, PO Box 5371, Seattle, WA 981452, United States of America
[11]Quantitative & Applied Ecology Group, School of BioSciences, University of Melbourne, Parkville, VIC 3010, Australia

*Corresponding author:
Simon I. Hay
Email: sihay@uw.edu
Phone: +1 206 8972878

Supplementary figure 1: Methods overview. Schematic overview of the methods representing input data, analyses, intermediate outputs and final outputs. Yellow fever = YF.

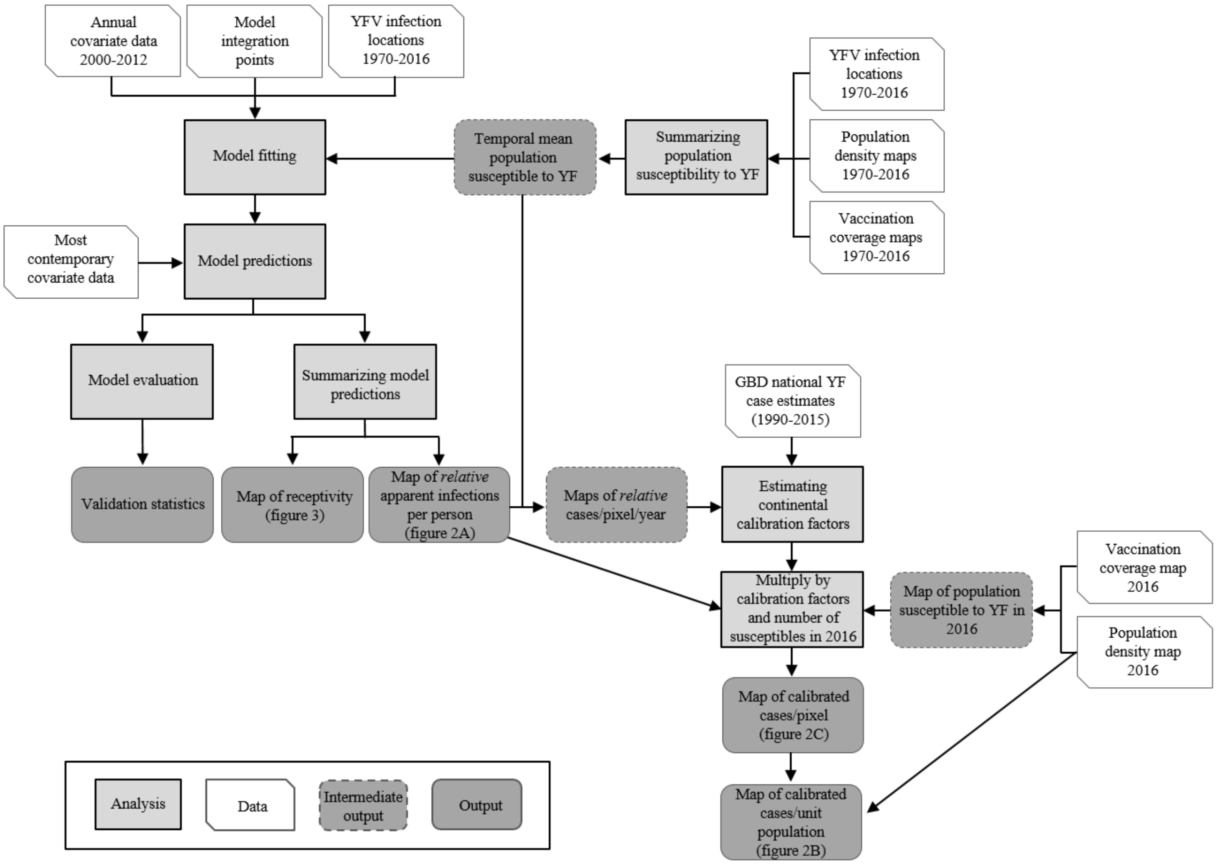

Assembly of the geo-referenced YFV human infection occurrence dataset

We assembled a database of locations where at least one symptomatic human infection of YFV has been reported in any given year. This information was extracted from a variety of sources including online sources, peer-reviewed literature and WHO reports. The data from online sources were collated by the automated HealthMap system (http://www.healthmap.org), as described elsewhere.[1, 2] HealthMap alerts for YF were obtained for the years from 2006 to 2015, and then checked manually for validity. To collate the data from published literature, ISI Web of Science and PubMed were searched using the term "yellow fever" for all articles up until April 1st 2016. This returned 5,387 records. The article abstracts were reviewed and for those containing possible geographical information on YFV infection, the full text was obtained (725 articles).

Location information regarding laboratory confirmed YFV infections in humans was extracted from each peer-reviewed article or report (n=602) according to previously established protocols.[3, 4] Serological evidence of infection in healthy individuals was not included in the database due to uncertainty regarding time and site of infection and the patient's YF vaccination history. If an article reported multiple cases in the same location (same 5×5 km grid square) within the same calendar year, this was recorded as a single YF occurrence record. Spatial coordinates were assigned to each reported site of infection using a combination of information from the article or report and online gazetteers such as Google Maps, OpenStreetMap and Geonames. For locations smaller than 5×5 km in area (termed 'points'), only the latitude and longitude in decimal degrees were recorded. The remaining sites, referred to as 'polygons', were assigned an administrative area code (eg, for a province or district) which were linked to shapefiles from the Food and Agricultural Organization (www.fao.org/geonetwork/), for first and second order administrative units, or Global Administrative Areas (http://www.gadm.org/), for third and fourth order administrative units.

Of the 1154 records in the final dataset: 402 were diagnosed via PCR-based or other genetic detection techniques; 444 were diagnosed via serological evidence; and the remaining 308 were reported as confirmed cases without specifying the diagnostic test used. The effectiveness of serological diagnostic techniques is limited, so in order to check whether the predicted distribution of YF risk was sensitive to the inclusion/exclusion of occurrence data diagnosed via PCR-based or other genetic detection techniques, we also fitted a model without these data. We found that the predicted distribution was not sensitive to the inclusion/exclusion of the PCR-based /genetic detection occurrence data (Supplementary figure 2).

Supplementary figure 2: Model sensitivity to inclusion/exclusion of PCR diagnosed occurrence data. A. Model fitted with the full occurrence dataset (copied from manuscript figure 2A). **B.** Fitted model excluding occurrence data diagnosed by PCR-based or other genetic detection techniques.

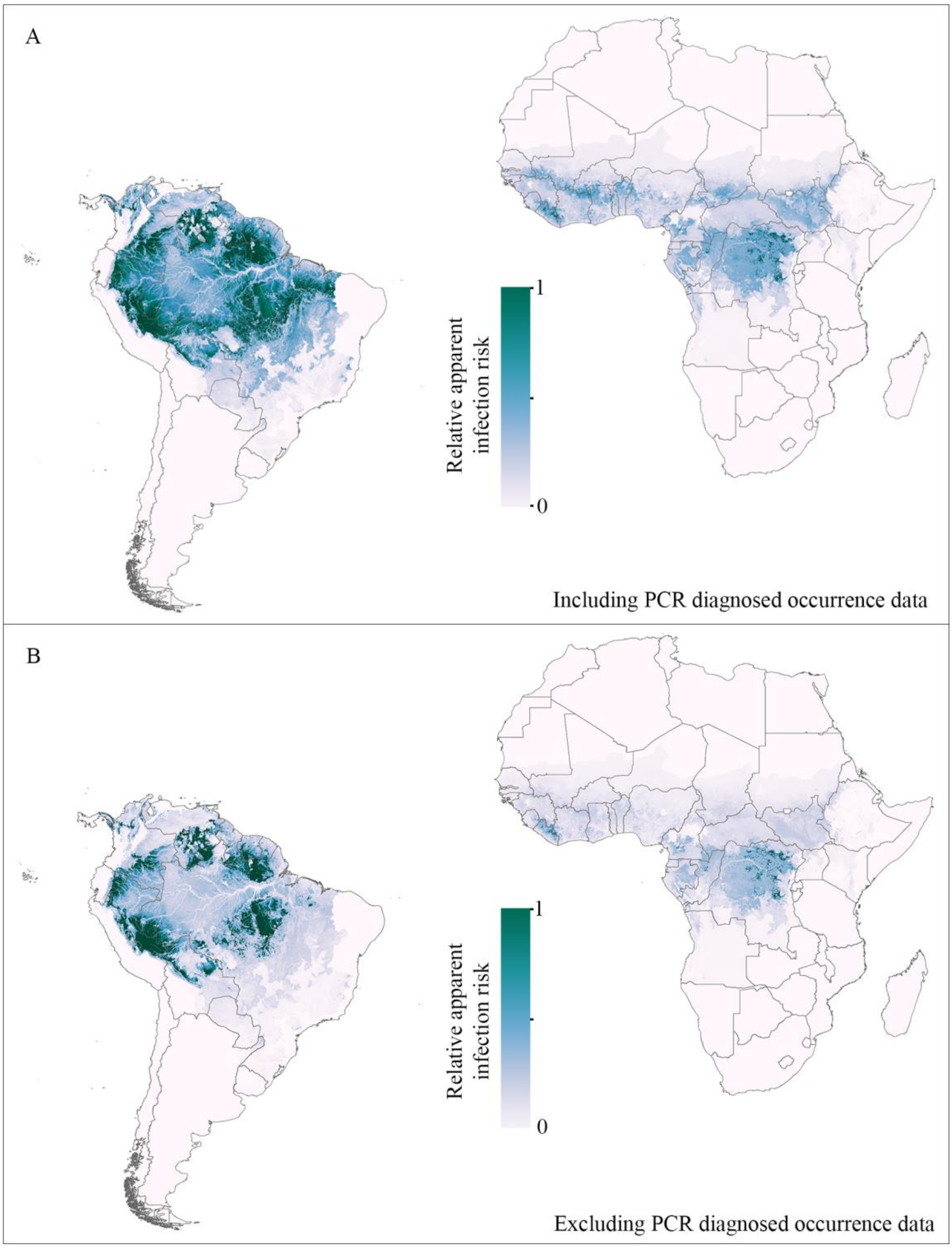

Summarising YF vaccination coverage and population density data

Accounting for the effects of YF vaccination in our temporally static model of YF infection risk required estimates of average vaccination coverage across the study time period. To calculate this temporal average, we used estimates for every five years from 1970 to 2016 of the proportion of the population across all age cohorts who had ever received a YF vaccine for each district within every at-risk country. Details on how these vaccination coverage estimates were calculated can be found elsewhere.[5] Since rates of YF reporting varied over the study time period, we calculated the weighted average, with weights representing the proportion of YF occurrences reported in each five year time period.

Shearer and colleagues[5] generated three alternative vaccination coverage estimates, each corresponding to one of three targeting scenarios for historical campaigns. 1) *Untargeted, unbiased*: assuming vaccination history was not taken into account and all individuals had an equal chance of receiving a vaccine regardless of their previous vaccination status. 2) *Targeted*: assuming that vaccination history was taken into account and only non-vaccinated individuals were targeted by immunisation campaigns. 3) *Untargeted, biased*: assuming that vaccination history was not taken into account and that previously vaccinated individuals were more likely to be targeted inadvertently ie, due to demographic biases in vaccination uptake. In the *untargeted, biased* scenario, for each vaccination campaign it was assumed that all previously vaccinated individuals received vaccines before any unvaccinated individuals. This scenario produced maximally conservative estimates of vaccination coverage, while estimates from the *targeted* scenario were maximally optimistic.

For model fitting, we used average vaccination coverage estimates based on the *untargeted, unbiased* vaccination-targeting scenario. Maps of YF vaccination coverage, averaged across the 47-year study time period (1970-2016), and for 2016, are provided in Supplementary figure 3. We also calculated average human population density over the study time period using a combination of human population datasets. Human population density surfaces at 5×5 km resolution from 2000 to 2015 were generated using a combination of WorldPop[6] and Gridded Population of the World[7] data. These two datasets were mosaicked together, with WorldPop data used in preference where they overlapped, as it is a more spatially dis-aggregated dataset. United Nations World Population Prospects[8] data was then used to generate projections of human population density for every five years from 1970 to 1995, and for 2016.

Supplementary figure 3: Average and contemporary yellow fever vaccination coverage. A. Temporal mean vaccination coverage from 1970 to 2016, calculated using time series estimates by Shearer and colleagues.[5] **B.** Estimated vaccination coverage for 2016. Both maps are based on the *untargeted, unbiased* vaccination-targeting scenario.

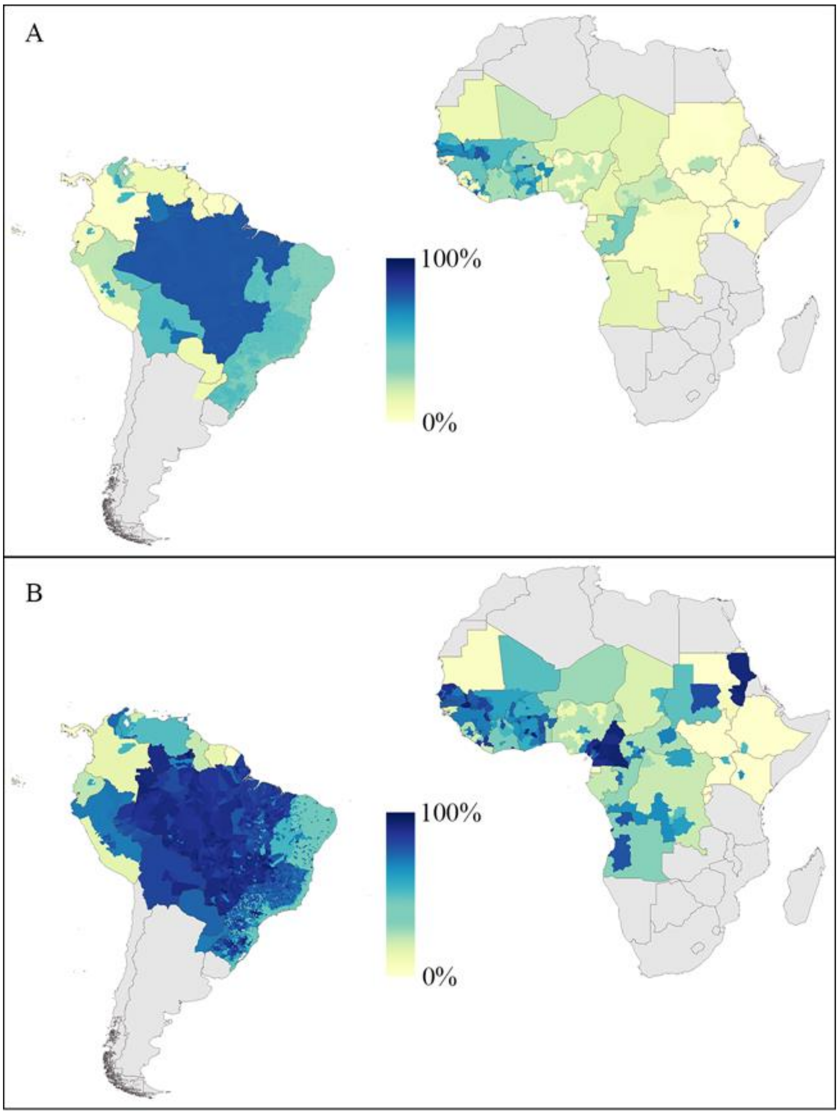

Model explanatory covariates

The environmental covariates included measures of the mean values for each pixel for tasseled cap wetness (TCW) and enhanced vegetation index (EVI), both related to surface moisture. These were derived from NASA's moderate resolution imaging spectrometer (MODIS) satellite imagery[9, 10]. They were computed from the original 1×1 km dataset and gap-filled using the Weiss algorithm to model missing data caused by cloud cover.[11]

We also included a data surface of temperature suitability for persistent established *Aedes aegypti* populations.[12] It has been shown that an index of temperature suitability that incorporates the relationships between adult mosquito longevity and temperature can be a better predictor of mosquito-borne disease risk than temperature alone.[13]

Land cover covariates were derived from the International Geosphere-Biosphere land cover classification available within the MODIS MCD12Q1 dataset at a 500×500 m spatial resolution.[14] The 500×500 m categorical datasets were summarized to produce a value for proportional land cover of either 'urban and built up', 'evergreen broadleaf forests', or 'cropland and natural vegetation mosaics' within the larger 5×5 km grid cells. Annual data surfaces were calculated using data for each year from 2001 to 2012. Additional information on covariates derived from satellite imagery and their subsequent processing, is provided elsewhere.[13] The elevation covariate was derived from the Shuttle Radar Topography Mission (SRTM) dataset,[15] with the original 90 m spatial resolution data summarized to produce a 5×5 km surface.[13]

Finally we included a fine-scale predictive species distribution map for the suspected non-human primate reservoirs of YFV. Details on the construction of this covariate are provided below. Since the original data for our covariates came from a variety of sources, all covariate grids were standardized to ensure identical spatial resolution, extent and boundaries.

Supplementary figure 4: Covariates used to predict the distribution of yellow fever infection in humans. **A.** Relative habitat suitability for vector *Aedes aegypti*. **B.** Index of temperature suitability for *Ae. aegypti*. Grey = unsuitable. **C.** Enhanced vegetation index. **D.** Tasselled cap wetness - a measure of surface moisture.

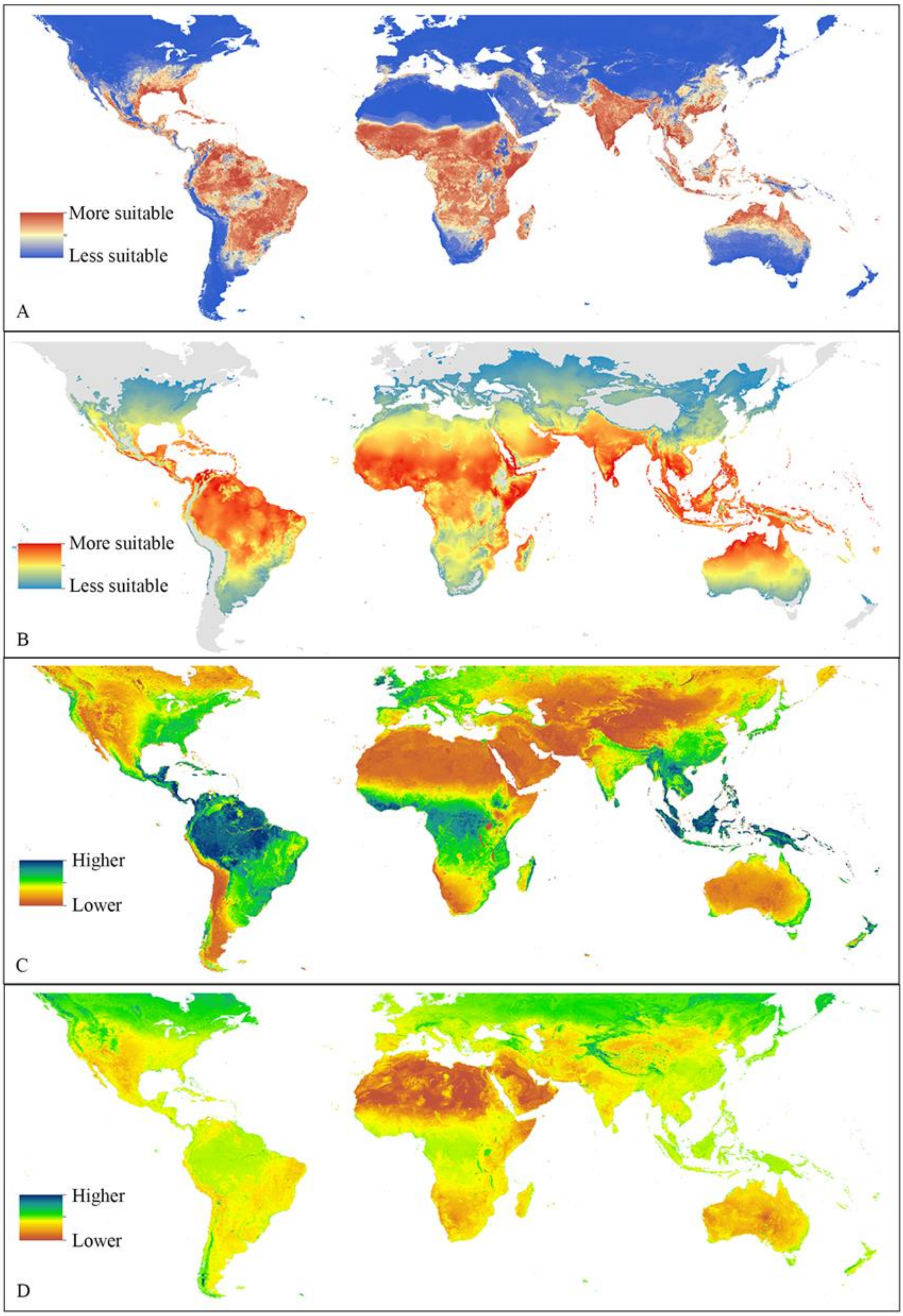

Supplementary figure 5: Land cover and elevation covariates used to predict the distribution of yellow fever infection in humans. **A.** Elevation. **B-D.** Proportional cover for 2012 of urban and built-up landscapes, evergreen broadleaf forest, and croplands and natural vegetation mosaics, respectively.

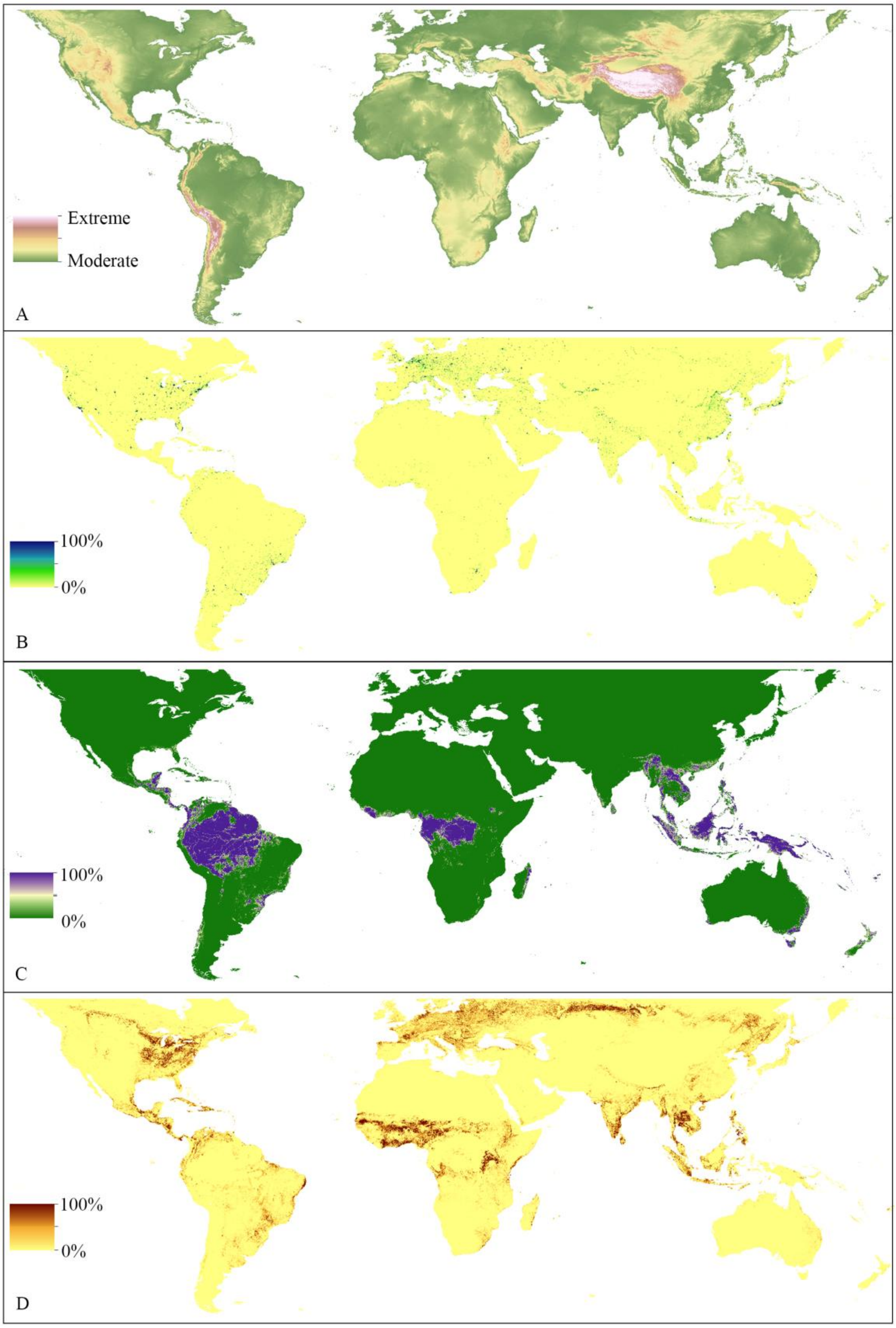

Mapping the distribution of suspected non-human primate reservoirs
We predicted the distribution of suspected reservoir species of non-human primates using a Bernoulli niche modeling approach previously applied to the mapping of disease reservoir and vector species.[16] The Poisson point process niche modeling approach described below allows for the incorporation of other factors (such as vaccination coverage), and clearer interpretation of the model output in certain circumstances, but at the cost of greater computational intensity, especially in model validation. Since there were no such factors to account for here, and the output was only being used as a covariate (thus the absolute value was not important), the Bernoulli approach was preferable.

In Africa, and Central and South America, various species of non-human primate have been implicated as natural hosts and reservoirs of YFV.[17] The role of a particular host species in the epidemiology of YF is dependent on their ability to circulate YFV at sufficiently high titers and to provide subsequent transmission of virus to vectors above minimum threshold levels.[18] When infected with YFV, non-human primates on the African continent develop viremias sufficient to infect mosquitoes, but they are relatively resistant to the pathogenic effects of the virus. Conversely, non-human primates in Central and South America are highly susceptible to YFV infection and develop severe and often fatal disease. Epizootics involving the deaths of these non-human primate species can serve as a warning to nearby human populations of YF risk. The virus moves slowly from monkey group to neighboring monkey group in large epizootic waves that leave behind a few surviving, immune individuals.[17, 19]

To determine which non-human primate species should be included in our analysis, we searched PubMed on February 11th 2017, using the search terms (yellow[All Fields] AND (“fever” [MeSH Terms] OR “fever”[All Fields])) AND (reservoir[All Fields] OR (“primates”[MeSH Terms] OR “primates”[All Fields] OR “primate”[All Fields]) OR host[All Fields]) without any language or date restrictions. This search returned 3,830 records. Article abstracts were reviewed, and the full text was obtained for those articles containing possible evidence implicating a non-human primate species as a reservoir host for YFV (99 articles). Information was extracted from 61 of 99 full text articles.

Due to the short duration of viremia (2-5 days) and lack of clinical disease, most studies of African sylvatic YF transmission, involved either serological prevalence studies of wild monkeys,[20-27] or laboratory experiments testing for sufficient levels of viremia following inoculation with the virus.[28-30] Virus isolation (or positive antibody tests) from sera or tissue samples of diseased or deceased wild monkeys during active searches for epizootics were frequently reported in studies conducted in Central and South America.[31-40] In some cases, transmission from monkey-to-monkey via a mosquito vector was demonstrated, under laboratory conditions.[41, 42]

We included all non-human primate species where naturally acquired infection with YFV had been confirmed by serological or PCR-based techniques, and/or they had demonstrated sufficient levels of viremia post-inoculation with YFV under laboratory conditions. The following non-human primate species of Africa and South and Central America met this criteria: *Callicebus ornatus,*[42] *Cercocebus albigena*,[25, 26, 43] *Chlorocebus aethiops*,[44] *Colobus guereza/abyssinicus*,[26, 30, 43, 45, 46] *C. polykomos,*[20, 25] *Cercopithecus mitis*,[25, 26, 47] *C. mona*,[25, 27, 43] *C. nictitans*,[23, 27] *C. neglectus,*[27] *C. ihoesti,*[48] *C. ascanius,*[22, 24, 26, 27, 43] *C. diana,*[20] *Erythrocebus patas*,[21, 27, 49, 50] *Galago senegalensis*,[45, 51, 52] *G. crassicaudatus* (*Otolemur crassicaudatus*),[47, 52] *Papio hamadryas/cynocephalus*,[27, 46] *P. papio*,[21] *P. doguera*,[25, 26] and *Piliocolobus badius*,[20, 26, 53] of Africa, and *Alouatta guariba*,[32, 33, 54] *A. palliata*,[55-58] *A. caraya*,[32, 33, 36, 54, 59, 60] *A. seniculus*,[39, 40, 61, 62] *A. belzebul,*[34] *Ateles paniscus*,[63] *A. fusciceps*,[64] *A. geoffroyi,*[56, 64] *Aotus trivirgatus*,[44] *A. zonalis*,[64] *Cebus apella*,[65-67] *C. capucinus*,[56, 66] *Marikina geoffroyi*,[57, 64] *Pithecia pithecia*,[61, 62] *Saguinus midas*,[61, 62] *S. oedipus*,[44] and *Saimiri scieureus*[42, 68] of Central and South America. Occurrence data were not available for *Marikina geoffroyi*, *Cercopithecus ihoesti*, or *Papio doguera*, so these species were subsequently not included in our analysis.

Since one of our aims was to predict the receptivity for YF transmission in regions outside its current risk zones, we also mapped the distribution of non-human primate species of Asia that have demonstrated susceptibility to YFV infection under laboratory conditions. These included *Macaca fascicularis*, *M. sinica* and *M. mulatta*.[68-70] In particular, *Macaca mulatta* (rhesus macaques) infected with YFV exhibit severe disease that generally models the course of the disease in humans and serve as a highly suitable animal model for YFV research.[71]

Separate species distribution models were carried out to predict the distribution of potential reservoir

species on each continent. Occurrence data for each species were retrieved from the Global Biodiversity Information Facility (GBIF) (www.gbif.org) totaling 931, 1193, and 820 records for African, Latin American, and Asian species, respectively. Records falling outside the International Union for Conservation of Nature (IUCN) (www.iucn.org) range maps for each species were excluded from the analysis. Standard practice in the species distribution mapping field is to supplement occurrence records with background or pseudo-absence points to represent areas where the species has not been reported, and then model the presence/background label for each point as a draw from a Bernoulli likelihood. It has been demonstrated that predictive accuracy of presence/background species distribution models can be improved by selecting background data with similar spatial bias to the occurrence records. The aim is to achieve the same environmental bias in both datasets, so the resulting model should identify suitable environments for the species within the sampled space, rather than just areas that experience more sampling, such as easily accessible locations. The spatial distribution of survey effort can be estimated by collecting occurrence records for a broad biological group, where the sampling methods are similar to those used for the target species.[72] For this study, we used the location of all other mammal surveys held by GBIF as background data. This resulted in 5661, 14,274, and 743, background points for the African, Latin American and Asian reservoir models, respectively.

Ten explanatory covariates were included in each model, including elevation, enhanced vegetation index (mean), land surface temperature (daytime mean), and tasseled cap wetness (mean) as well as six proportional land cover classes: cropland and natural vegetation mosaic, woody savannas, savannas, urban and built-up, evergreen broadleaf forest and deciduous broadleaf forest. Further details on the source and construction of each covariate data surface are provided in the 'Model explanatory covariates' section. Since the collection date was not available for the vast majority of occurrence records, land cover data surfaces for 2012 were linked to all occurrence records.

We fitted an ensemble of 100 Bernoulli boosted regression tree models (sub-models) to each continent dataset using the gbm3_2.2 R package.[73] Each sub-model was trained to a separate bootstrap dataset randomly sampled with replacement from the complete occurrence/background dataset for that continent. The model algorithm hyper-parameters were set to the following values: cross-validation folds=10, tree complexity=3, learning rate=0.0005, and bag fraction=0.5. We followed the procedure used in Moyes and colleagues[16] for mapping the distribution of macaque reservoir species of *Plasmodium knowlesi* malaria, and further details on model fitting can be found there.

To generate the final prediction maps, a mean predicted value of environmental suitability was calculated across the model ensemble. Prior to inclusion in the YF disease model, each prediction was clipped to the joint species IUCN range maps for that continent. Finally, the predictions for each continent were joined to produce a single reservoir covariate data surface (Supplementary figure 6). Standard deviation values were also calculated for each pixel across the model ensembles. These outputs were then clipped and joined to produce a map of reservoir model uncertainty (Supplementary figure 8).

To evaluate the predictive performance of each continent model, we calculated the area under the receiver operator curve (AUC) statistic, ie, the area under a plot of the true positive rate versus false positive rate, reflecting the ability to discriminate between occurrence and background records.[74] The overall statistic for each model was calculated as the mean of the AUCs for each sub-model in the model ensemble. The models demonstrated moderate to high predictive performance, with overall AUC statistics of 0.754 (± 0.008), 0.806 (± 0.006), and 0.771 (± 0.012) for the African, Latin American and Asian species distribution models, respectively. The ROC curves are also plotted in Supplementary figure 7.

Supplementary figure 6: Non-human primate reservoir distribution map. Composite habitat suitability maps for suspected and potential non-human primate reservoir species for Latin America, Africa and Asia (**A**), the locations of occurrence data (**B**) and background data (**C**) used to generate each continent map.

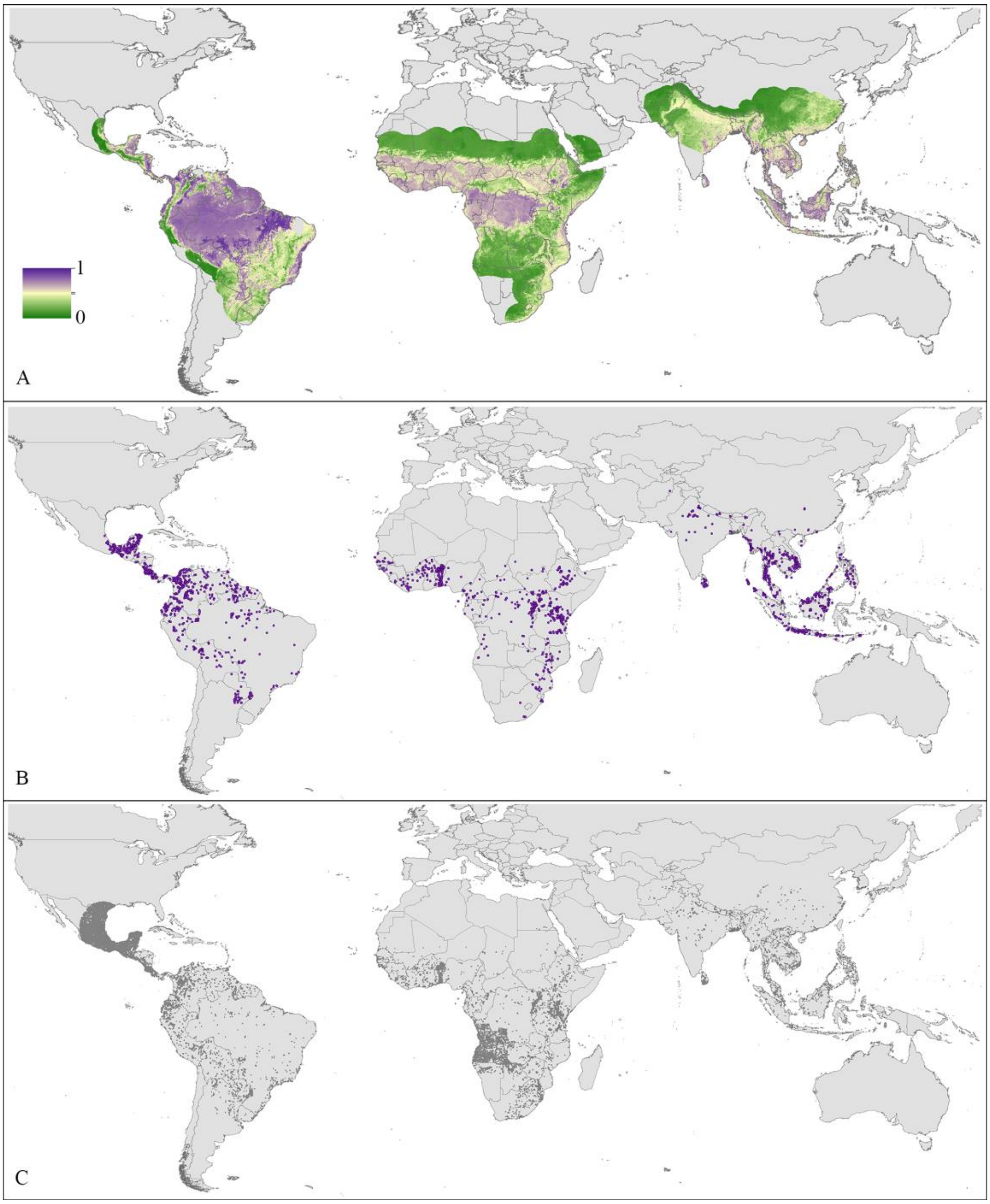

Supplementary figure 7: ROC curves. Plots of the ROC curves for suspected and potential non-human primate reservoir distribution models generated for each continent.

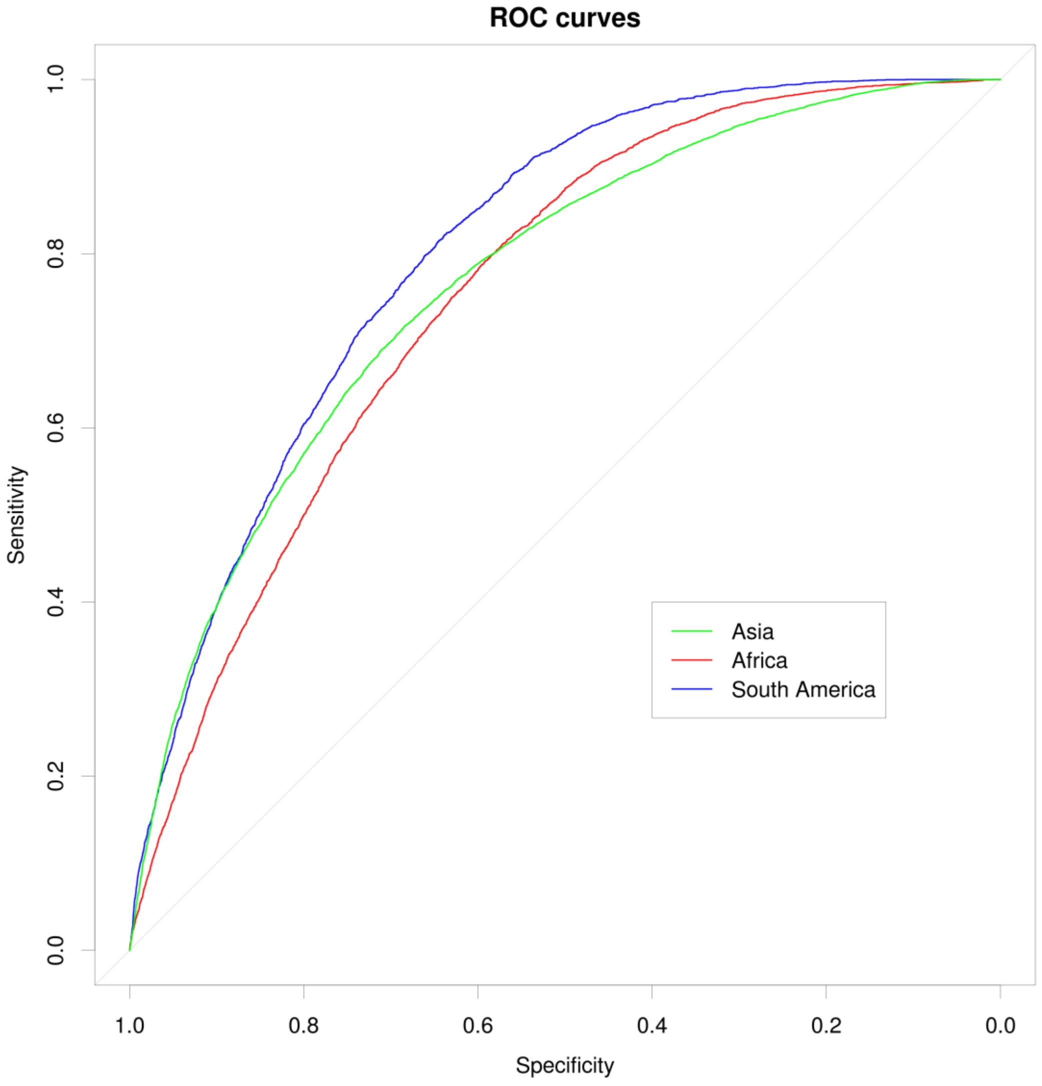

Supplementary figure 8: Map of reservoir distribution model uncertainty. Composite map of estimate pixel-wise uncertainty in predictions of habitat suitability for suspected and potential non-human primate reservoirs of YF. Uncertainty estimates are based on standard deviation values calculated for each pixel across the model ensemble for each continent.

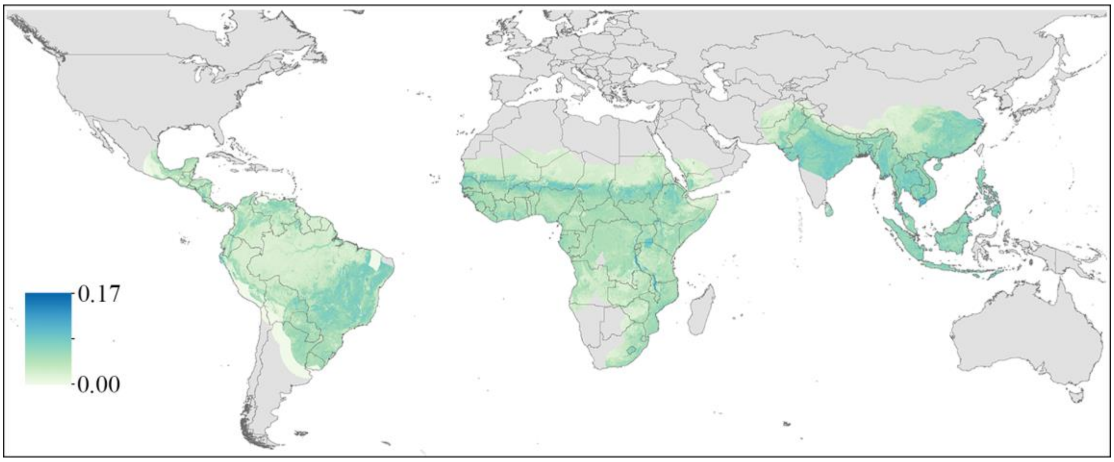

Modelling YF occurrences as an inhomogeneous Poisson point process
Species distribution modelling (SDM) has been widely applied in epidemiology to produce predictive maps of reservoir, vector and disease distributions from reports of disease occurrence.[75] Previous applications of SDM for disease mapping have typically used background or pseudo-absence points to represent areas where the disease has not been reported, and then used a binomial likelihood, even though the data does not meet the assumptions of this model.[76] Recently point process models have been proposed in the ecological SDM literature as a more appropriate tool for SDM of occurrence-only data. These models directly consider the data as a set of point locations where a species has been recorded as occurring.[76-78] The point process framework therefore resolves a number of important issues for occurrence-only SDM including interpretation, selection of pseudo-absence points, accounting for biases and checking of model assumptions.[76, 78]

Inhomogeneous Poisson point process models
The inhomogeneous Poisson point process (hereafter PPM) is among the simplest and most well studied point processes that can be used to analyse occurrence-only data. Given a set of locations at which a species (or disease) has been observed in geographic region A, we can characterise the point process from which the locations are drawn via an intensity function over space λ(s) – the expected number of observations per unit area. Conditional on this intensity function, the locations of the points are assumed to be independent of each other, and the total number of points in A is thus a Poisson random variable with mean:

$$(1) \quad \Lambda_{\mathrm{A}} = \int_{\mathrm{A}} \lambda(s) ds$$

We may also assume that the intensity surface of the point process λ(s) varies spatially and via some relationship with spatially varying environmental conditions x(s). The expected number of points per unit area can then be modelled as a function of covariates measured throughout the study region A.[78]

The most common approach to fitting a PPM is to maximise its likelihood function. Evaluating this likelihood requires calculation of the integral of the intensity function over A – the expected number of occurrence points. In general the likelihood cannot be calculated directly, but can be approximated efficiently by evaluating a weighted Poisson likelihood at a set of quadrature points in space. These quadrature points are superficially similar to background points in a presence-background SDM analysis, but have clear mathematical interpretation under the PPM framework. Hence prior to model fitting, we need to decide how quadrature points will be chosen, and how to calculate the quadrature weights at each quadrature and presence point. Frameworks for selecting quadrature points, along with examples, are provided in a number of recent publications.[76-78]

A useful implication of the PPM approach is that if it is assumed that the observed locations represent *all* disease events in the area, then the intensity λ(s) represents the expected abundance of events. Most of the time this is not the case as not all occurrences of disease are reported, and the fraction reported is rarely known. However in these cases the intensity can instead be interpreted as being directly proportional to abundance, provided any spatial variation in sampling effort has been taken into account (eg, *via* offsets or covariates included in the model). This explicit link with abundance enables integration of distribution modelling with fundamental epidemiological models that consider immunity and metrics of disease dynamics.

Model description
We fitted a PPM to a dataset of YF disease reports using a boosted regression tree model to characterise the intensity function. By explicitly accounting for human population density, vaccination rate, and variable reporting effort this model estimated the number of apparent YFV infections per person to within a constant of proportionality at each 5×5 km grid square in the study region.

We assumed that the number of YF disease reports in the any given area A within the study region follows a Poisson distribution:

$$(2) \quad N_A^{+} \sim Poisson\ (\Lambda_{\mathrm{A}})$$

We further assumed that the set of locations (points) arose from an intensity function $\lambda(s)$ comprising the product of three independent processes: the number of apparent infections per person, the number of individuals susceptible to infection and the proportion of infections reported:

$$(3) \qquad \Lambda_A = \int_A \lambda(s)\, ds = \int_A I(s)P(s)R(s) ds$$

Where *I(s)* gives the expected number of reported apparent infections over the study period at location s, *P(s)* gives the susceptible population density at point location s, and *R(s)* gives the number of reports per infection (reporting rate) at location s. Our aim was to infer the spatial process *I(s)* governing incidence, so we sought to specify *P(s)* and *R(s) a priori*.

We directly estimate the population density of susceptible individuals $P(s)$ as the product of the overall population density and the fraction of the total population who had not received a vaccine against YFV averaged over the study period. The model does not take into account the proportion of the population with immunity from natural infections. We also assumed that the YF vaccine provides lifelong 100% protection, which is supported by recent WHO recommendations.[79]

Whilst the *absolute* reporting rate process *R(s)* could not be estimated reliably, spatial variation in reporting rates can be inferred from the distribution of reports generally.[72, 80] We therefore factorised $R(s) = k\, r(s)$ where r(s) is a probability distribution giving the relative reporting rate (and integrating to one over the study area), and k is a constant of proportionality which we cannot estimate *a priori*. Rather than inferring $r(s)$ directly, we followed Philips and colleagues[72] in collating a set z of n coordinates giving the locations of reports of human infectious diseases and treated these as random samples from $r(s)$. Specifically z were drawn from a database of locations within the study area where at least one case of human infectious disease has been reported. This approach assumes that YF and all the diseases in this database are subject to a common spatial variation in reporting rate. Further details on the assembly of the database of disease reports are provided in the 'Selection of model integration points' section below.

This sample from $r(s)$ enables us to represent Eq. (3) via a Monte-Carlo approximation during model fitting. Writing the Monte Carlo approximation to a function over a probability distribution as:

$$(4) \qquad \int_A f(s)\pi(s)\, ds \approx \frac{1}{n}\sum_i^n f(z_i); \quad z_i \sim \pi(s)$$

and substituting $f(s) = k\, I(s)P(s)$ and $\pi(s) = r(s)$, we have:

$$(5) \qquad \Lambda_A = \int_A k\, I(s)P(s)\, r(s) ds \approx \frac{1}{n}\sum_i^n k\, I(z_i)P(z_i)$$

We fitted a PPM to the point pattern of YF reports, with a BRT model for the intensity function $\lambda(s)$. We approximated the PPM likelihood via the Monte Carlo sum above, using the Poisson likelihood and the set of locations z as integration points, following the approach previously proposed for modelling PPMs in GLM software using numerical quadrature.[78, 81, 82] The value of the susceptible population density process $P(z_i)$ was provided for each quadrature point as an offset value, leaving the BRT to estimate only the function $k\, I(s)$. Equation (3) assumes a linear relationship between the number of YFV infections and the number of susceptible individuals, thus the values of $I(s)$ predicted by the BRT model represent the incidence of apparent infections in susceptible individuals over the study time period, to within an unknown constant of proportionality k.

Model fitting
We fitted an ensemble of Poisson point process boosted regression tree (BRT) models using the 'gbm3' R package.[73] The BRT approach has the ability to fit complex nonlinear responses including high-dimensional interactions between explanatory variables, has been shown to have high predictive accuracy and has been previously applied to disease distribution mapping. Boosted regression trees combine two algorithms, regression trees (which repeatedly split the data into two groups using a randomly selected predictor variable for each split) and boosting (which additively fits trees to the data, gradually prioritizing poorly modelled data to produce a set of trees that maximally reduce the loss

function), to examine and quantify the relationship between explanatory variables and the response data.

Rather than exclusively using synoptic (averaged across time) covariate values for each of the occurrence locations irrespective of the occurrence date, we incorporated annual land cover data surfaces from 2001 to 2012 (data were not available for years outside this time period). Occurrence data collected between 2001 and 2012 were matched with covariate values for the relevant year; 66% of data points fell within this time period. Covariate values for 2001 were used for occurrence data prior to this date and covariate values for 2012 were used for post-2012 data. Final predictions were made to the most contemporary covariate values available. Land cover data were not available to match with occurrence data reported prior to 2001, and we were therefore unable to account for changes to the landscape (such as from urbanization, agriculture and deforestation) that occurred from 1970 to 2000.

To increase the robustness of model predictions and quantify model uncertainty, we fitted an ensemble of 100 BRT models (sub-models), each trained to a separate bootstrap dataset. We applied a spatial block bootstrapping procedure where the study space was stratified into 160 regular grid squares (spatial blocks) and each bootstrap dataset consisted of all occurrence data and model integration points falling within a set of blocks randomly sampled with replacement from the complete set. Each cross-validation fold consisted of data and integration points falling within a non-contiguous sample of spatial blocks, generated for the bootstrapping procedure. We used a Poisson likelihood to approximate the PPM likelihood (specifying the Poisson density as a loss function) and integration weights were applied to each model integration point, equaling the estimated number of individuals susceptible to YF infection at that location, and these were incorporated in the model as a logarithmic offset (passed *via* the offset argument). The algorithm hyper-parameters were set to the following values: cross-validation folds=10, tree complexity=3, learning rate=0.005, and bag fraction=0.75. The model was restricted so that YF transmission had a monotonic positive relationship with both habitat suitability and temperature suitability for *Ae. aegypti*. To incorporate uncertainty in the precise location of infection for polygon occurrence records, each bootstrap dataset randomly selected a 5×5 km pixel within each polygon, biased towards human population density, since more populous areas have a greater probability of reporting at least one case.

To generate the final prediction surface, a mean predicted value was calculated across the 100 sub-models for each 5×5 km pixel across the risk zones. Standard deviation values for each pixel were also calculated across the model ensemble to provide an estimate model uncertainty (figure 3).

Selection of model integration points

Eight thousand model integration points were drawn from a database of reports of human infectious diseases. The aim of this dataset was to reflect the spatial bias in reporting rates of YFV infections. A number of approaches for estimating spatial bias in occurrence data are described in the ecological literature, where datasets are similarly subject to spatial bias in survey effort. One approach is to use occurrence records for a broad biological group (such as birds or mosquitoes) to represent overall survey effort. Multiple factors are likely to impact disease reporting, including the strength of surveillance systems, treatment seeking behaviour, availability of accurate diagnostics, accessibility to healthcare facilities, and disease prevalence, etc. We felt that the best way of encompassing these multiple issues in the integration dataset was to include reports from a broad range of human infectious diseases.

One drawback of this approach is that certain locations will overall experience higher rates of disease in general than others. In other words, the distribution of the integration points will actually represent a combination of spatial variation in reporting rates (what we want to incorporate in the model) and spatial variation in the number of diseases that could be reported (what we do not want to incorporate in the model). However, since no global-scale, spatially-resolved maps of disease reporting rates are available, we believe this approach is the best practical solution to accounting for these biases.

Reports of human infectious diseases were collated from four publicly available datasets: 1) for falciparum and vivax malaria,[83] 2) the leishmaniases,[4] 3) dengue,[3] and 4) for numerous diseases (full list below) via the web-based system HealthMap, which automatically collates infectious disease reports from informal online data sources.[1, 2] The HealthMap records were collected from 2006 to 2014. This yielded 13387 geo-referenced reports from within the study region, after removing any reports falling within the same 5×5 km pixel. To avoid generating a set of model integration points

biased towards any one disease, sampling was biased towards diseases with lower counts and diseases with less than 20 reports were grouped before sampling.

Reports of the following diseases were collated via HealthMap: aeromonas and marine vibrio infection, anthrax, ascariasis, aspergillosis, avian influenza virus subtype H5N1, Bolivian haemorrhagic fever, botulism, brucellosis, campylobacteriosis, chikungunya, chlamydia infections, cholera, clostridium difficile colitis, common cold, Crimean-Congo haemorrhagic fever, cryptosporidiosis, cysticercosis, dengue, diphtheria, dracunculiasis (Guinea worm), Eastern equine encephalitis, Ebola virus disease, enterovirus infection, Escherichia coli diarrhoea, filariasis – bancroftian, giardiasis, glanders, hepatitis A, hepatitis B, hepatitis C, hepatitis E, histoplasmosis, HIV/AIDS, hookworm, infectious mononucleosis or EBV infection, influenza, Lassa fever, legionellosis, leishmaniasis – cutaneous, leishmaniasis – visceral, leprosy, leptospirosis, listeriosis, Marburg virus disease, measles, melioidosis, meningitis (viral), monkey pox, mumps, onchocerciasis, ornithosis, *P. falciparum* malaria, *P. malariae* malaria, *P. ovale* malaria, *P. vivax* malaria, parvovirus B19 infection, pediculosis, pertussis, plague, poliomyelitis, Q fever, rabies, respiratory syncytial virus infection, Rift Valley fever, Rocky Mountain spotted fever, rotavirus infection, rubella, salmonellosis, SARS, scabies, scarlet fever, schistosomiasis – haematobium, schistosomiasis – mansoni, shigellosis, St Louis encephalitis, syphilis, taeniasis, toxocariasis, toxoplasmosis, trachoma, trichinosis, trichuriasis, trypanosomiasis – African, trypanosomiasis – American, tuberculosis, typhoid and enteric fever, varicella, Venezuelan equine encephalitis, West Nile fever, and zika.

Supplementary figure 9: Model integration points. Location of integration points for yellow fever disease model fitting, representing intensity of disease case reporting.

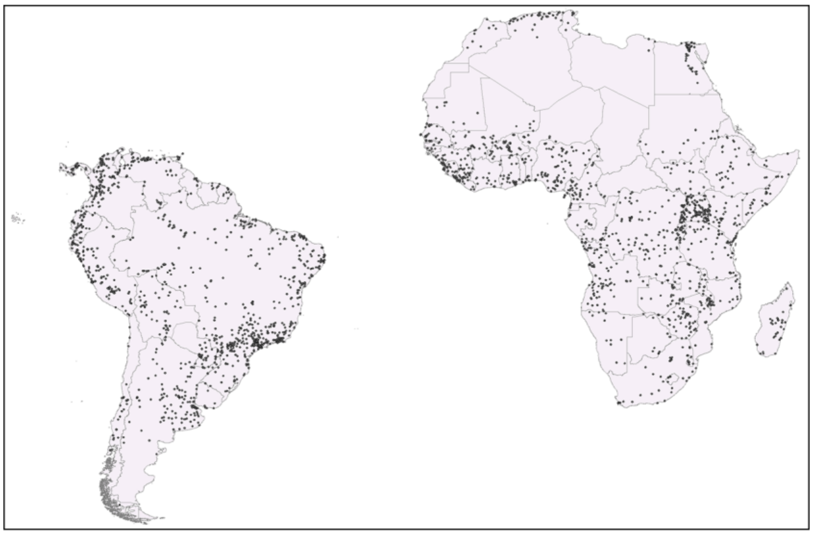

Model validation
The model's predictive performance was evaluated using spatially stratified ten-fold cross-validation and calculating the predictive deviance of the model; the mismatch between the predicted number of occurrence records and the number observed. We calculated McFadden's pseudo-R squared which is the ratio of the log likelihood of the full model (with covariates) and the log likelihood of the null model (without covariates). The ratio of the likelihoods indicates the level of improvement offered by the full model over the null (or intercept) model.[84] This ratio was calculated for each cross-validation fold model of each bootstrap sub-model and averaged across folds and sub-models, resulting in a bootstrapped estimate of the model's predictive capacity.

Validation statistics for the model ensemble indicated that model performance was high, with 92% of out-of-sample deviance explained by the model.

Most influential model covariates
The main predictors for YFV infection risk were the following: proportional cover of land classified as urban and built-up, temperature suitability for *Ae. aegypti*, habitat suitability for suspected non-human primate reservoir species, and enhanced vegetation index. Marginal effect plots for each of these covariates are included as Supplementary Figure 10.

Supplementary figure 10: Model marginal effort plots for the most influential covariates. The black line represents the mean marginal effect and grey envelopes the associated 95% quantiles. The mean relative contribution is displayed in the top left corner of each plot. The most influential predictors were defined as those whose relative influence was greater than 100/total number of covariates (since the sum of the relative influence values is 100). Marginal effect plots visualise the effect of each variable on the response after averaging the effects of all other variables.

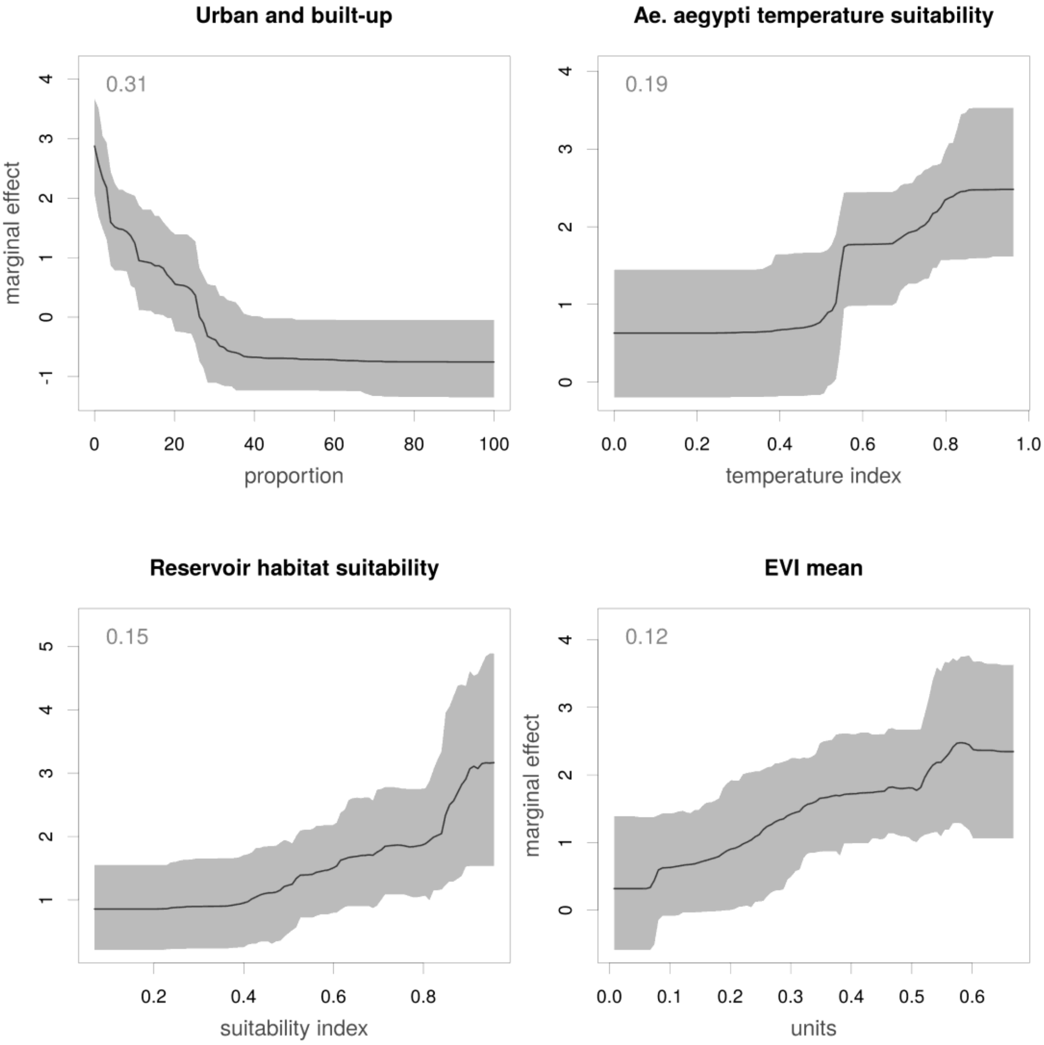

Model calibration

We calibrated our estimate of the *relative* number of YF cases using the average annual number of cases estimated by the Global Burden of Disease Study (GBD)[85] to obtain an estimate of the annual number of YF cases per grid cell, ie, we used a continental-scale estimate of incidence to estimate the average case detection rate, which is not identifiable from presence-only data alone. The GBD mean estimates (as well as lower and upper bounds) of national incidence were available for every five years from 1990 to 2015. We calculated the average annual number of YF cases predicted by GBD for each country over this time period. Using our map of relative annual YF cases (based on the average number of individuals susceptible to YFV infection over the study time period), we summed the total number of cases estimated by the model for each country. Three separate generalized linear models were then fitted to each of the three GBD estimated case numbers (mean, lower and upper), with our model estimated case numbers included as a logarithmic offset, and continent included as a fixed effect. Using the fitted model, we predicted calibration factors for each continent, 155.15 (lower bound=44.27, upper bound=405.99) for Africa, and 38.95 for South America (lower bound=10.32, upper bound=108.20). The mean calibration factors were then applied to the model output.

Whilst spatial variation in disease reporting rates was accounted for in model fitting through the selection of model integration points, case detection rates were assumed to be constant over space and time when calculating continental calibration factors to apply to the raw model output (figure 2A). However, we might expect that reporting rates have improved over time, and were therefore lower in the 1970s and 1980s. Since the calibration factors were calculated using GBD estimates from 1990 to 2015, and the relative average rate of apparent YF infections was calculated using data from 1970 to 2016, absolute incidence (figures 2B and C) may be underestimated due to inadequate correction.

Supplementary figure 11: For each country, the average number of cases estimated by GBD (from 1990 to 2015) plotted against the number of estimated cases after applying continental calibration factors. Upper and lower bounds are represented by black horizontal and vertical lines around the mean (blue dots). The line y=x is plotted in red.

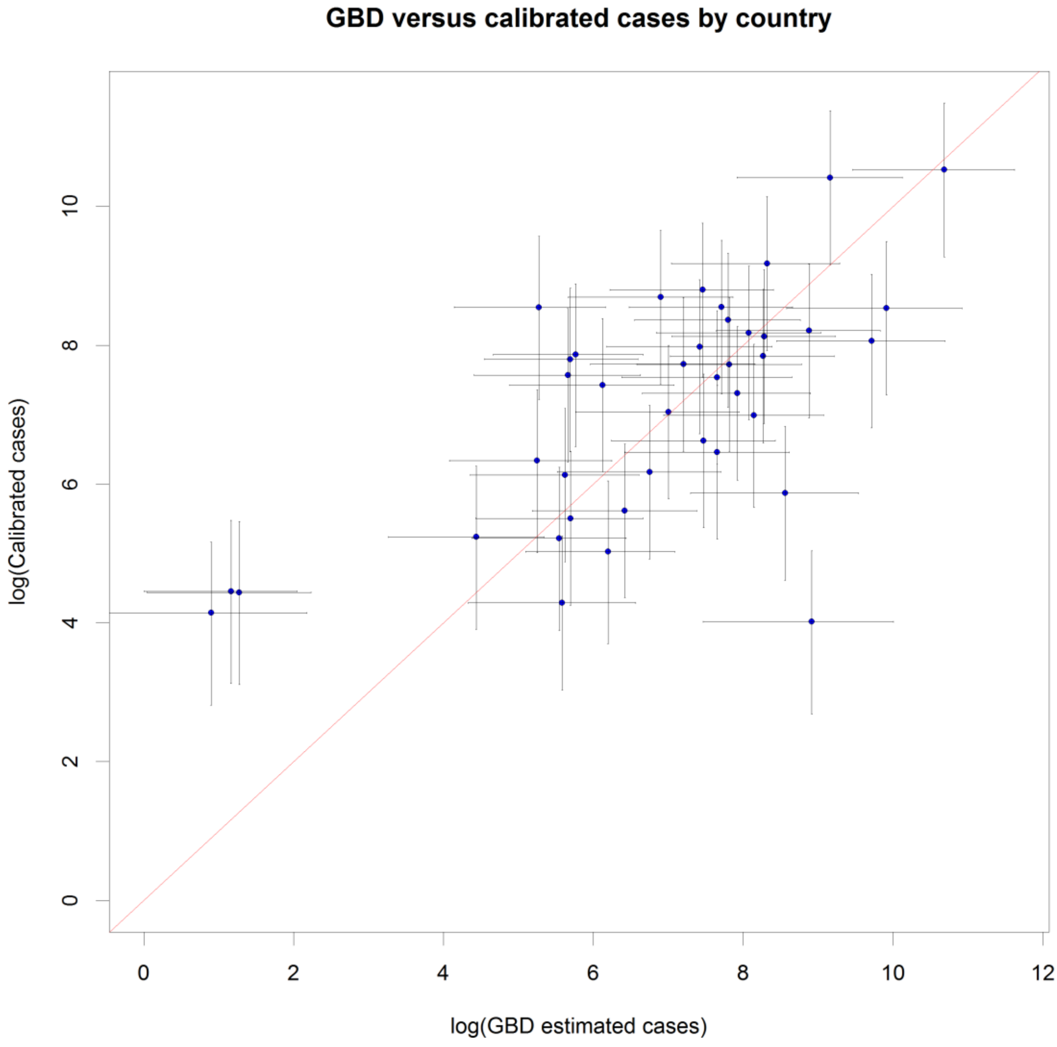

References

1. Freidfeld C, Mandl K, Reis B, Brownstein J. Healthmap: Global infectious disease monitoring through automated classification and visualization of Internet media reports. *Med Inform Assoc* 2008; **15**: 150–7.
2. Patching HMM, Hudson LM, Cooke W, Garcia AJ, Hay SI, Roberts M, et al. A supervised learning process to validate online disease reports for use in predictive models. *Big Data* 2015; **3**: 230–7.

3. Messina JP, Brady OJ, Pigott DM, Brownstein JS, Hoen AG, Hay SI. A global compendium of human dengue virus occurrence. *Sci Data* 2014; **1**: 140004.
4. Pigott DM, Golding N, Messina JP, Battle KE, Duda KA, Balard Y, et al. Global database of leishmaniasis occurrence locations, 1960–2012. *Sci Data* 2014; **1**: 140036.
5. Shearer FM, Moyes CL, Pigott DM, Brady OJ, Marinho F, Deshpande A, et al. Global yellow fever vaccination coverage from 1970 to 2016: an adjusted retrospective analysis. *Lancet Infect Dis*: http://dx.doi.org/10.1016/S473-3099(17)30419-X.
6. WorldPop. Gridded Population Distributions, 2015. http://www.worldpop.org.uk/ (accessed January, 2016). .
7. Center for International Earth Science Information Network – CIESIN – Columbia University, United Nations Food + Agriculture Programme – FAO, Centro Internacional de Agricultura Tropical – CIAT. Gridded Population of the World, Version 4 (GPWv4): Population Count Grid. Palisades, NY: NASA Socioeconomic Data and Applications Center (SEDAC); 2005.
8. United Nations. World Population Prospects, the 2015 Revision. https://esa.un.org/unpd/wpp/Download/Standard/Population/ (accessed October 13, 2016).
9. Lobser S, Cohen W. MODIS tasselled cap: land cover characteristics expressed through transformed MODIS data. *Int J Remote Sens* 2007; **28**: 5079–101.
10. Huete A, Justice C, Van Leeuwen W. MODIS vegetation index (MOD13). Algorithm theoretical basis document. 1999.
11. Weiss DJ, Atkinson PM, Bhatt S, Mappin B, Hay SI, Gething PW. An effective approach for gap-filling continental scale remotely sensed time-series. *ISPRS J Photogram Remote Sens* 2014; **98**: 106–18.
12. Brady OJ, Golding N, Pigott DM, Kraemer MUG, Messina JP, Reiner Jr RC, et al. Global temperature constraints on *Aedes aegypti* and *Ae. albopictus* persistence and competence for dengue virus transmission. *Parasit Vectors* 2014; **7**: 338.
13. Weiss DJ, Mappin B, Dalrymple U, Bhatt S, Cameron E, Hay SI, et al. Re-examining environmental correlates of *Plasmodium falciparum* malaria endemicity: a data-intensive variable selection approach. *Malar J* 2015; **14**: 68.
14. Friedl M, Sulla-Menashea D, Tanb B, Schneiderc A, Ramankuttyd N, Sibleya A, et al. MODIS Collection 5 global land cover: algorithm refinements and characterization of new datasets. *Remote Sens Environ* 2010; **114**: 168–82.
15. Farr TG, Rosen PA, Caro E, Crippen R, Duren R, Hensley S. The Shuttle Radar Topography Mission. *Rev Geophys* 2007; **45**: RG2004.
16. Moyes CL, Shearer FM, Huang Z, Wiebe A, Gibson HS, Nijman V, et al. Predicting the geographical distributions of the macaque hosts and mosquito vectors of *Plasmodium knowlesi* malaria in forested and non-forested areas. *Parasit Vectors* 2016; **9**: 242.
17. Hanley K, Monath T, Weaver S, Rossi S, Richman R, Vasilakis N. Fever versus fever: the role of host and vector susceptibility and interspecific competition in shaping the current and future distributions of the sylvatic cycles of dengue virus and yellow fever virus. *Infect Genet Evol* 2013; **19**: 292–311.
18. Vainio J, Cutts F. Yellow fever. Geneva: World Health Organization, 1998.
19. Rodhain F. The role of monkeys in the biology of dengue and yellow fever. *Comp Immunol Microbiol Infect Dis* 1991; **14**: 9–19.
20. Findlay GM, Maccallum FO. Yellow fever immune bodies in the blood of African primates. *Trans R Soc Trop Med Hyg* 1937; **31**: 103–6.
21. Traoré-Lamizana M, Fontenille D, Zeller HG, Mondo M, Diallo M, Adam F, et al. Surveillance for yellow fever virus in eastern Senegal during 1993. *J Med Entomol* 1996; **33**: 760–5.
22. Simpson DI, Haddow AJ, Williams MC, Woodall JP. Yellow fever in central Uganda, 1964. Part IV. Investigations on blood-sucking diptera and monkeys. *Trans R Soc Trop Med Hyg* 1965; **59**: 449–58.
23. Monath TP, Lee VH, Wilson DC, Fagbami A, Tomori O. Arbovirus studies in Nupeko forest, a possible natural focus of yellow fever virus in Nigeria. I. Description of the area and serological survey of humans and other vertebrate hosts. *Trans R Soc Trop Med Hyg* 1974; **68**: 30–8.
24. Kirya BG, Okia NO. A yellow fever epizootic in Zika Forest, Uganda, during 1972: Part 2: Monkey serology. *Trans R Soc Trop Med Hyg* 1977; **71**: 300–3.
25. Haddow AJ, Smithburn KC, Mahaffy AF, Bugher JC. Monkeys in relation to yellow fever in Bwamba County, Uganda. *Trans R Soc Trop Med Hyg* 1947; **40**: 677–700.

26. Haddow AJ, Dick GW, Lumsden WH, Smithburn KC. Monkeys in relation to the epidemiology of yellow fever in Uganda. *Trans R Soc Trop Med Hyg* 1951; **45**: 189–224.
27. Mathiot CC, Herve VM, Georges AJ. Antibodies to haemorrhagic fever viruses and to selected arboviruses in monkeys from the Central African Republic. *Trans R Soc Trop Med Hyg* 1990; **84**: 732–3.
28. Henderson BE, Cheshire PP, Kirya GB, Lule M. Immunologic studies with yellow fever and selected African group B arboviruses in rhesus and vervet monkeys. *Am J Trop Med Hyg* 1970; **19**: 110–8.
29. Woodall JP. The reaction of a mangabey monkey (*Cercocebus galeritus agilis* Milne-Edwards) to inoculation with yellow fever virus. *Ann Trop Med Parasitol* 1968; **62**: 522–7.
30. Woodall JP, Dykes JR, Williams MC. The reaction of a species of colobus monkey to inoculation with yellow fever virus. *Ann Trop Med Parasitol* 1968; **62**: 528–35.
31. Trapido H, Galindo P. The investigation of a sylvan yellow fever epizootic on the north coast of Honduras, 1954. *Am J Trop Med Hyg* 1955; **4**: 665–74.
32. Holzmann I, Agostini I, Areta JI, Ferreyra H, Beldomenico P, Di Bitetti MS. Impact of yellow fever outbreaks on two howler monkey species (*Alouatta guariba clamitans* and *A. caraya*) in Misiones, Argentina. *Am J Primatol* 2010; **72**: 475–80.
33. de Almeida MA, Dos Santos E, da Cruz Cardoso J, da Fonseca DF, Noll CA, Silveira VR, et al. Yellow fever outbreak affecting Alouatta populations in southern Brazil (Rio Grande do Sul State), 2008–2009. *Am J Primatol* 2012; **74**: 68–76.
34. Vasconcelos PF, Rosa AP, Rodrigues SG, Rosa ES, Monteiro HA, Cruz AC, et al. Yellow fever in Pará State, Amazon region of Brazil, 1998–1999: entomologic and epidemiologic findings. *Emerging Infectious Diseases* 2001; **7**: 565–9.
35. Sallis ESV, de Barros VLRS, Garmatz SL, Fighera RA, Graça DL. A case of yellow fever in a brown howler (*Alouatta fusca*) in Southern Brazil. *J Vet Diagn Invest* 2003; **15**: 574–6.
36. Moreno ES, Rocco IM, Bergo ES, Brasil RA, Siciliano MM, Suzuki A, et al. Reemergence of yellow fever: detection of transmission in the State of Sao Paulo, Brazil, 2008. *Rev Soc Bras Med Trop* 2011; **44**: 290–6.
37. Moreno ES, Spinola R, Tengan CH, Brasil RA, Siciliano MM, Coimbra TLM, et al. Yellow fever epizootics in non-human primates, São Paulo state, Brazil, 2008-2009. *Revista do Instituto de Medicina Tropical de São Paulo* 2013; **55**: 45–50.
38. Leal SG, Romano APM, Monteiro RV, Melo CBd, Vasconcelos PFdC, Castro MBd. Frequency of histopathological changes in Howler monkeys (*Alouatta sp*.) naturally infected with yellow fever virus in Brazil. *Revista da Sociedade Brasileira de Medicina Tropical* 2016; **49**: 29–33.
39. Auguste AJ, Lemey P, Bergren NA, Giambalvo D, Moncada M, Moron D, et al. Enzootic transmission of yellow fever virus, Venezuela. *Emerg Infect Dis* 2015; **21**: 99–102.
40. Anderson CR, Downs WG. The isolation of yellow fever virus from the livers of naturally infected red howler monkeys. *Am J Trop Med Hyg* 1955; **4**: 662–4.
41. Ross RW, Gillett JD. The cyclical transmission of yellow fever virus through the grivet monkey, *Cercopithecus aethiops centralis* Neumann, and the mosquito *Aedes (Stegomyia) africanus* Theobald. *Ann Trop Med Parasitol* 1950; **44**: 351–6.
42. Bates M, Roca-García M. An experiment with neurotropic yellow fever virus in *Saimiri* monkeys and *Haemagogus* mosquitoes. *Am J Trop Med Hyg* 1946; **s1–26**: 607-12.
43. McCrae AW, Kirya BG. Yellow fever and Zika virus epizootics and enzootics in Uganda. *Trans R Soc Trop Med Hyg* 1982; **76**: 552-62.
44. Bates M, Roca-Garcia M. Experiments with various Colombian marsupials and primates in laboratory cycles of yellow fever. *Am J Trop Med Hyg* 1946; **26**: 437–53.
45. Lumsden WH, Buxton AP. A study of the epidemiology of yellow fever in West Nile District, Uganda. *Trans R Soc Trop Med Hyg* 1951; **45**: 53–78.
46. Sérié C, Andral L, Poirier A, Lindrec A, Neri P. Etudes sur la fièvre jaune en Ethiopie. 6. Etude épidémiologique. *Bull World Health Organ* 1968; **38**: 879–84.
47. Haddow AJ. A review of the results of yellow fever protection-tests on the sera of primates f rom Kenya. *Ann Trop Med Parasitol* 1952; **46**: 135–43.
48. Kading RC, Borland EM, Cranfield M, Powers AM. Prevalence of antibodies to alphaviruses and flaviviruses in free-ranging game animals and nonhuman primates in the greater Congo basin. *J Wildl Dis* 2013; **49**: 587–99.
49. Felsenfeld O, Wolf RH, Dutta NK. Serological Responses of Patas Monkeys (*Erythrocebus patas*) to Vaccination Against Cholera and Yellow Fever. *Proc Soc Exp Biol Med* 1973; **143**: 548–50.

50. Deubel V, Pailliez JP, Cornet M, Schlesinger JJ, Diop M, Diop A, et al. Homogeneity among Senegalese strains of yellow fever virus. *Am J Trop Med Hyg* 1985; **34**: 976–83.
51. Taylor RM, Haseeb MA, Work TH. A regional reconnaissance on yellow fever in the Sudan; with special reference to primate hosts. *Bull World Health Organ* 1955; **12**: 711–25.
52. Haddow AJ, Ellice JM. Studies on bush-babies (*Galago spp*.) with special reference to the epidemiology of yellow fever. *Trans R Soc Trop Med Hyg* 1964; **58**: 521–38.
53. Findlay GM, Stefanopoulo GJ, Davey TH, Mahaffy AF. Yellow fever immune bodies in the blood of African animals. *Trans R Soc Trop Med Hyg* 1936; **29**: 419–24.
54. Laemmert HW, Jr., Kumm HW. The susceptibility of howler monkeys to yellow fever virus. *Am J Trop Med Hyg* 1950; **30**: 723–31.
55. Boshell J, Bevier GA. Yellow fever in the lower Motagua Valley, Guatemala. *Am J Trop Med Hyg* 1958; **7**: 25-35.
56. Galindo P, Derodaniche E. Surveillance for sylvan yellow fever activity in Panama (1957-1961). *Am J Trop Med Hyg* 1964; **13**: 844-50.
57. De Rodaniche E. Survey of primates captured in Panama, R. P. during the years 1952-1956 for protective antibodies against yellow fever. *Am J Trop Med Hyg* 1957; **6**: 835–9.
58. Vargas-Mendez O, Elton NW. Naturally acquired yellow fever in wild monkeys of Costa Rica. *Am J Trop Med Hyg* 1953; **2**: 850–63.
59. Morales MA, Fabbri CM, Zunino GE, Kowalewski MM, Luppo VC, Enría DA, et al. Detection of the mosquito-borne flaviviruses, West Nile, Dengue, Saint Louis Encephalitis, Ilheus, Bussuquara, and Yellow Fever in free-ranging black howlers (*Alouatta caraya*) of Northeastern Argentina. *PLOS Negl Trop Dis* 2017; **11**: e0005351.
60. Tranquilin MV, Lehmkuhl RC, Maron A, Silva LR, Ziliotto L, Seki MC, et al. First report of yellow fever virus in non-human primates in the State of Parana, Brazil. *Rev Soc Bras Med Trop* 2013; **46**: 522–4.
61. de Thoisy B, Dussart P, Kazanji M. Wild terrestrial rainforest mammals as potential reservoirs for flaviviruses (yellow fever, dengue 2 and St Louis encephalitis viruses) in French Guiana. *Trans R Soc Trop Med Hyg* 2004; **98**: 409–12.
62. de Thoisy B, Vogel I, Reynes JM, Pouliquen JF, Carme B, Kazanji M, et al. Health evaluation of translocated free-ranging primates in French Guiana. *Am J Primatol* 2001; **54**: 1–16.
63. Karesh WB, Wallace RB, Painter RL, Rumiz D, Braselton WE, Dierenfeld ES, et al. Immobilization and health assessment of free-ranging black spider monkeys (*Ateles paniscus chamek*). *Am J Primatol* 1998; **44**: 107–23.
64. De Rodaniche EC. Survey of live forest animals for protective antibodies against yellow fever in panama, R.P. . *Am J Trop Med Hyg* 1952; **1**: 789–95.
65. Downs WG. Epidemiological notes in connection with the 1954 outbreak of yellow fever in Trinidad, B.W.I. *Yellow fever, a symposium in commemoration of Carlos Juan Finlay, 1955 Paper 4* 1955.
66. Pinheiro FP, Travassos da Rosa AP, Moraes MA. An epidemic of yellow fever in Central Brazil, 1972–1973. II. Ecological studies. *Am J Trop Med Hyg* 1981; **30**: 204–11.
67. Lima MA, Romano-Lieber NS, Duarte AMRdC. Circulation of antibodies against yellow fever virus in a simian population in the area of Porto Primavera Hydroelectric Plant, São Paulo, Brazil. *Rev Inst Med Trop S Paulo* 2010; **52**: 11–6.
68. Price WH, Casals J, O'Leary W. Studies on the sequential immunization against group B arboviruses in squirrel monkeys, cynomolgus monkeys, rhesus monkeys, and chimpanzees. *Am J Trop Med Hyg* 1974; **23**: 118–30.
69. Stokes A, Bauer JH, Hudson NP. The transmission of yellow fever to Macacus rhesus. 1928. *Rev Med Virol* 2001; **11**: 141–8.
70. Levenbook IS, Pelleu LJ, Elisberg BL. The monkey safety test for neurovirulence of yellow fever vaccines: the utility of quantitative clinical evaluation and histological examination. *J Biol Stand* 1987; **15**: 305–13.
71. Julander JG. Animal models of yellow fever and their application in clinical research. *Curr Opin Virol* 2016; **18**: 64–9.
72. Phillips S, Dudik M, Elith J, Graham C, Lehmann A, Leathwick J, et al. Sample selection bias and presence-only distribution models: implications for background and pseudo-absence data. *Ecol Appl* 2009; **19**: 181–97.
73. Ridgeway G with contributions from others (2015) gbm: generalized boosted regression models. R package version 2.2. Available from: http://CRAN.R-project.org/package=gbm.
74. Fleiss J, Levin B, Paik M. Statistical methods for rates and proportions. Hoboken, New Jersey: John Wiley & Sons; 2003.

75. Purse B, Golding N. Tracking the distribution and impacts of diseases with biological records and distribution modelling. *Biol J Linnean Soc* 2015; **115**: 664–7.
76. Wharton D, Shepherd L. Poisson point process models solve the 'pseudo-absence problem' for presence-only data in ecology. *Ann Appl Stat* 2010; **4**: 1383–402.
77. Aarts G, Fieberg J, Matthiopoulos J. Comparative interpretation of count, presence–absence and point methods for species distribution models. *Methods Ecol Evol* 2012; **3**: 177–87.
78. Renner IW, Elith J, Baddeley A, Fithian W, Hastie T, Phillips SJ, et al. Point process models for presence-only analysis. *Methods Ecol Evol* 2015; **6**: 366–79.
79. World Health Organization. Vaccines and vaccination against yellow fever. WHO Position Paper – June 2013. *Weekly Epidemiological Record* 2013; **88**: 269–84.
80. Fithian W, Elith J, Hastie T, Keith DA. Bias correction in species distribution models: pooling survey and collection data for multiple species. *Methods in Ecology and Evolution* 2015; **6**: 424–38.
81. Berman M, Turner T. Approximating Point Process Likelihoods with GLIM. *Appl Statist* 1992; **41**: 31–8.
82. Simpson D, Illian J, Lindgren F, Sorbye S, Rue H. Going off grid: computationally efficient inference for log-Gaussian Cox processes. *Biometrika* 2016; **103**: 49–70.
83. Moyes CL, Temperley WH, Henry AJ, Burgert CR, Hay SI. Providing open access data online to advance malaria research and control. *Malar J* 2013; **12**: 161.
84. Long JS. Regression model for categorical and limited dependent variables. Thousand Oaks: Sage Publications; 1997.
85. Global, regional, and national incidence, prevalence, and years lived with disability for 310 diseases and injuries, 1990-2015: a systematic analysis for the Global Burden of Disease Study 2015. *Lancet* 2016; **388**: 1545-602.
